# A cladistically based reinterpretation of the taxonomy of two Afrotropical tenebrionid genera *Ectateus* Koch, 1956 and *Selinus* Mulsant & Rey, 1853 (Coleoptera, Tenebrionidae, Platynotina)

**DOI:** 10.3897/zookeys.415.6406

**Published:** 2014-06-12

**Authors:** Marcin Jan Kamiński

**Affiliations:** 1 Museum and Institute of Zoology, Polish Academy of Sciences, Wilcza 64, 00-679 Warsaw, Poland

**Keywords:** Africa, ecoregions, cladistics, identification key, new genus, taxonomy, Pedinini

## Abstract

On the basis of a newly performed cladistic analysis a new classification of the representatives of two Afrotropical tenebrionid genera, *Ectateus* Koch, 1956 and *Selinus* Mulsant & Rey, 1853 *sensu* Iwan 2002a, is provided. *Eleoselinus* is described as a new genus. The genus *Monodius*, previously synonymized with *Selinus* by Iwan (2002), is redescribed and considered as a separate genus. Following new combinations are proposed: *Ectateus calcaripes* (Gebien, 1904), *Monodius laevistriatus* (Fairmaire, 1897), *Monodius lamottei* (Gridelli, 1954), *Monodius plicicollis* (Fairmaire, 1897), *Eleoselinus villiersi* (Ardoin, 1965) and *Eleoselinus ursynowiensis* (Kamiński, 2011). Neotype for *Ectateus calcaripes* and lectotypes for *E. crenatus* (Fairmaire, 1897), *E. ghesquierei* Koch, 1956 and *Monodius malaisei malaisei* Koch, 1956 are designated to fix the taxonomic status of these taxa. The following synonymies are proposed: *Selinus monardi* Kaszab, 1951 and *Ectateus latipennis* Koch, 1956 with *E. crenatus* (Fairmaire, 1897). Identification keys are provided to all known species of *Ectateus* *sensu novum*, *Eleoselinus*, *Monodius* and *Selinus* *sensu novum*.

## Introduction

Pursuant to the classification of the family Tenebrionidae presented by Bouchard et al. (2005, 2011) Platynotina Mulsant & Rey, 1853 is one of the eight subtribes within the tribe Pedinini Eschscholtz, 1829. At present Platynotina consists of over 60 genera distributed in Afrotropical, Indomalayan, Nearctic and Neotropical realms (Iwan 2002b; Kamiński 2013c; Kamiński and Raś 2012).

According to the results of a cladystic analysis performed by Iwan (2002a), *Ectateus* Koch, 1956 and *Selinus* Mulsant & Rey, 1853 are the members of the platynotoid evolutionary lineage within the subtribe Platynotina Mulsant & Rey, 1853. The representatives of both genera are distributed in the western parts of Central Africa (Iwan 2004a).

The current taxonomic concept of the genus *Ectateus* was proposed by Iwan (2002a) and modified by Kamiński and Raś (2011) to: circular depressions on the lateral sides of clypeus and genae, pronotum with anterior angles distinctly protruding anteriorly, elytral humeri not protruding outwards, apical part of epipleuron and fifth ventrite unbordered. The taxonomic concept of *Selinus* was also established by Iwan (2002a) and is as follows: upper edge of elytral base fused with humerus, anterior pronotal angles distincly protruding anteriad, short metasternum and bursa copulatrix with two sacs. Unfortunately both of the above mentioned taxonomic concepts were based only on a few representatives of their genera. The preliminary study of the entomological material has shown that some of the representatives of *Ectateus* shares many morphological characters and distributional pattern with certain species of the *Selinus* and *vice versa*.

According to the results of a cladistic analysis performed by Iwan (2002a) *Ectateus* and *Selinus* are members of two sister clades. In the key to the genera of World Platynotina they are distinguished by the structure of 5^th^ abdominal ventrite (*Selinus* – with bordering or border interrupted; *Ectateus* – without bordering) (Iwan 2002a). Unfortunately, this feature is no longer relevant which may easily lead to misidentification (five of seven species of *Selinus* do not match this character). Additionally, *Ectateus* and *Selinus* shares some unique (within whole subtribe) morphological features (e.g. slender antennomeres, specific clavae structure) and similar distributional pattern (Iwan 2002a, 2002b, Kamiński and Raś 2011). All this suggests that both of the mentioned genera can be more closely related than it was implied by Iwan (2002a).

The aim of this paper was to test the monophyly of *Ectateus* and *Selinus* and propose a stable classification for the representatives of these genera.

## Material and methods

**Morphological studies.** The descriptive sequence used in this study is in accordance with Kamiński (2013b). Morphological terms follow Matthews et al. (2010); with additional specialized terms used for the male (Iwan 2001b, 2004b) and female genitalia (Banaszkiewicz 2006).

Measurements, taken using a filar micrometer, were as follows: width of anterior elytral margin (from humeral angle to scutellum); body length (from anterior margin of labrum to elytral apex); body width (maximum elytral width).

For examination of internal structures, specimens were dissected and whole abdomens were cleared in 10% cold potassium hydroxide overnight (Iwan 2000).

Images were taken using a Canon 1000D body with accordion bellows and Industar 61L/3 MC 50 mm f/2.8 lens, and with a Hitachi S-3400N SEM in MIIZ. Chosen SEM photographs were colored using Photoshop CS5.

**Entomological material.** This study was based on the material from the following collections:

BMNH Natural History Museum, London, Great Britain

HNHM Hungarian Natural History Museum, Budapest, Hungary

JFCS Julio Ferrer Collection, Haninge, Sweden

MHNG Muséum d’histoire naturelle de la Ville de Genève, Geneva, Switzerland

MIIZ Museum and Institute of Zoology, Polish Academy of Sciences, Warsaw, Poland

MNB Museum für Naturkunde, Germany, Berlin

MNHN Muséum National d’Histoire naturelle, Paris, France

MHNL Centre de Conservation des Collections, Muséum d’Histoire Naturelle, Lyon, France

MRAC Royal Museum for Central Africa, Tervuren, Belgium

RBINS Royal Belgian Institute of Natural Sciences, Brussels, Belgium

TMNH Ditsong National Museum of Natural History, Pretoria, Republic of South Africa

SMNS Staatliches Museum für Naturkunde, Stuttgart, Germany

ZMAS Zoological Museum, Academy of Sciences, Sankt Petersburg, Russia

**Phylogenetic analysis.** Based on the results of a comparative analysis of the morphology of available material, including the type specimens, I propose a following synonymy: *Selinus monardi* Kaszab, 1951 and *Ectateus latipennis* Koch, 1956 with *Ectateus crenatus* (Fairmaire, 1897). Also, I disagree with the synonymy of *Selinus calcaripes* Gebien, 1904 with *Ectateus curtulus* (Fairmaire, 1893) proposed by Koch (1956) and I propose to treat this taxon as a independent species – not as a synonym of *Ectateus curtulus*. For detailed information see the descriptions of these taxa included in the results section of this publication.

The operational taxonomic units (OTUs) representing the genus *Ectateus* consists of all (8) known species (considering above mentioned nomenclatural acts): *Ectateus crenatus* (Fairmaire, 1897), *Ectateus curtulus* (Fairmaire, 1893), *Ectateus ghesquierei* Koch, 1956, *Ectateus laevistriatus* (Fairmaire, 1897), *Ectateus lamottei* (Gridelli, 1954), *Ectateus modestus* (Fairmaire, 1887), *Ectateus ursynowiensis* Kamiński, 2011 and *Ectateus villiersi* Ardoin, 1965. Also, all (7) known species of *Selinus* were included in the phylogenetic analysis: *Selinus convexipennis* Gebien, 1904, *Selinus gravis* Koch, 1956, *Selinus malaisei* Koch, 1956, *Selinus medius* Fairmaire, 1897, *Selinus planus* (Fabricius, 1792), *Selinus plicicollis* Fairmaire, 1897 and *Selinus striatus* (Fabricius, 1794). The above mentioned taxa form the ingroup.

*Zidalus latipes* (Sahlberg, 1823) was used as the most distant outgroup on which the character polarization process was performed. According to Iwan’s (2002a) hypothesis the genus *Zidalus* Mulsant & Rey, 1853 is a sister clade to all afrotropical platynotoid genera.

*Lechius abacoides* (Fairmaire, 1902), *Pseudoselinus punctatostriatus* (Gerstaecker, 1854), *Upembarus upembaensis* Koch, 1956 were used to test the monophyly of the clade *Ectateus*+*Selinus*. According to the results of Iwan’s (2002a) cladistic analysis the genus *Lechius* Iwan, 1995 together with *Pseudoselinus* Iwan, 2002 and *Upembarus* Koch, 1956 form a sister clade to the *Ectateus* generic group (which includes *Ectateus* and *Selinus*). This hypothesis was supported by more recent studies (Iwan and Kamiński 2012, Kamiński 2012, Raś and Kamiński 2013).

The data matrix originated in Mesquite (Maddison and Maddison 2011). Parsimony analysis was conducted under equal weights in TNT (Goloboff et al. 2003). Most parsimonious tree was obtained by the “Implicit enumeration”. Jackknife support (absolute frequencies) was calculated with 36 removal probability using 2000 replicates. Consistency index (CI) and retention index (RI) were used to assess the fit of data to the cladograms (Farris 1989). The results were illustrated using WinClada (Nixon 2002).

**Species distribution.** The distribution of species was illustrated using DIVA-GIS version 7.5 (Hijmans et al. 2012). The raster layer used in Figs 41–44 was downloaded from naturalearthdata.com (“Made with Natural Earth. Free vector and raster map data”). The division of Afrotropical Realm into ecoregions was adopted after Olson et al. (2001).

## Results

**Character matrix.** A matrix of 40 characters was constructed for 20 operational taxonomic units (Table 1). Characters used for phylogenetic analyses have been treated as unordered. The missing data for *Ectateus curtulus* are caused by the fact that this species is only known from one specimen (holotype, female). The character states are presented in this section.

**Table 1.** Character matrix for the cladistic analysis of the species of *Ectateus* and *Selinus* (sensu Iwan 2002a), with selected outgroup taxa: *Zidalus latipes*, *Lechius abacoides*, *Pseudoselinus punctatostriatus*, *Upembarus upembaensis* (see also text).

Taxon / character	1	6	11	16	21	26	31	36
*Zidalus latipes*	00000	00000	01000	10000	00000	00100	00000	00000
*Lechius abacoides*	00000	10000	01000	00000	00000	00000	00000	00000
*Pseudoselinus punctatostriatus*	00000	01000	01000	00101	00000	01100	00000	00000
*Upembarus upembaensis*	00000	01000	01000	00100	00000	00100	00000	00000
*Ectateus crenatus*	00111	00110	10211	10011	11100	00001	00100	10001
*Ectateus curtulus*	0?010	00100	10211	00011	10100	00???	?????	??000
*Ectateus ghesquierei*	00111	00110	10211	00011	11100	00001	00100	10001
*Ectateus laevistriatus*	01001	10000	00101	10001	00000	10110	01011	01100
*Ectateus lamottei*	01001	10000	00101	00001	00000	10110	01011	01100
*Ectateus modestus*	01111	00110	10211	10011	10100	00001	00100	10000
*Ectateus ursynowiensis*	10000	00000	00100	01000	00101	00001	00100	00000
*Ectateus villiersi*	10000	00001	00100	01000	00001	00001	00100	00000
*Selinus calcaripes*	01111	00110	10211	10011	10000	00001	00100	10000
*Selinus convexipennis*	01001	10000	00101	00001	00000	10110	01010	01100
*Selinus gravis*	01000	10000	00100	00001	00000	10110	01010	01100
*Selinus malaisei*	01000	10000	00100	00000	00010	10110	11010	02100
*Selinus medius*	01000	10000	00100	00000	00010	10110	11010	02100
*Selinus planus*	01000	10001	00100	00001	00000	01100	00010	00010
*Selinus plicicollis*	01000	10000	00100	00000	00010	10110	11010	02100
*Selinus striatus*	01000	10001	00100	00001	00000	01100	00010	00010

**Head (characters 1–7)**

1. Anntenna: (0) slender, longer than pronotum; (1) robust, shorter than pronotum.

2. Antennomeres from 7 to 11: (0) widened, their width greater than the length; (1) elongated, their length greater than the width (Fig. 3).

**Figures 1–7.**
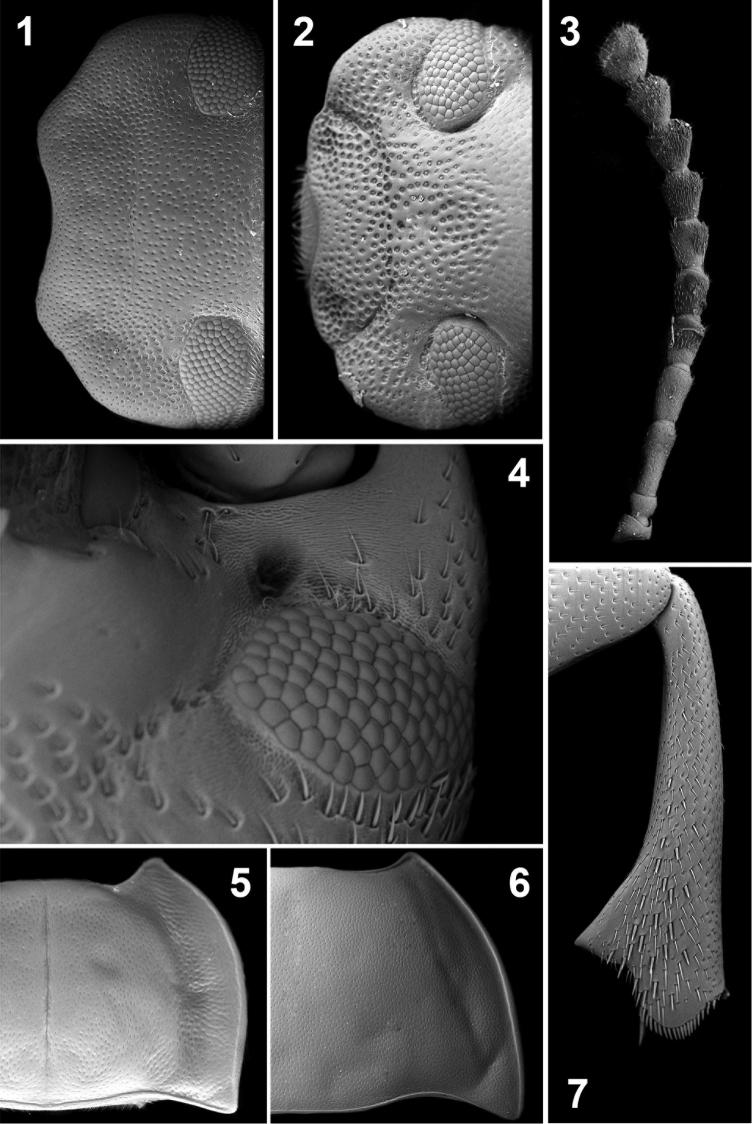
Head, dorsal view (**1, 2**), ventral view (**4**); antenna (**3**); pronotal disc (**5, 6**); mesotibia (**7**). *Ectateus calcaripes* (**3**), *Ectateus crenatus* (**2**), *Ectateus ghesquierei* (**5**), *Monodius medius* (**7**), *Monodius plicicollis* (**4**), *Selinus planus* (**1, 6**).

3. Circular depressions on the lateral sides of clypeus and genae: (0) absent (Fig. 1); (1) present (Fig. 2).

4. Fronto-clypeal suture: (0) fine (Fig. 1); (1) coarse, clearly visible (Fig. 2).

5. Indentation between frons and clypeus on the lateral edge: (0) shallow (Fig. 1); (1) deep (Fig. 2).

6. Anterior tentorial pit: (0) shallow; (1) deep, clearly visible (Fig. 4).

7. Anterior part of mentum: (0) not elongated; (1) elongated.

**Prothorax** (characters 8–18)

8. Anterior pronotal angles: (0) straight; (1) curved outwards (Fig. 5).

9. Lateral pronotal sides: (0) rounded; (1) sinusoidal (Fig. 5).

10. Pronotum: (0) widest at the middle (Fig. 5); (1) widest at the base (Fig. 6).

11. Pronotal margins: (0) not erected upwards; (1) strongly erected upwards (Raś and Kamiński 2013, Kamiński 2013c).

12. Ratio of prothorax width (tw) and pronotal disc height (dh): (0) < 5; (1) > 6 (Raś and Kamiński 2013, Kamiński 2013c).

13. Apophyseal depressions: (0) absent; (1) trapezoidal (Fig. 6); (2) rounded (Fig. 5).

14. Pronotal base: (0) the same width as elytral base; (1) narrower than elytral base.

15. Posterior pronotal angles: (0) not protruding towards elytra; (1) strongly protruding towards elytra.

16. Punctures on pronotal disc: (0) fine, the intervals between the punctures are greater than the 2 diameters of the puncture; (1) coarse, the intervals between the punctures are smaller than the diameter of the puncture.

17. Intercoxal process of prosternum: (0) flat or dented (Fig. 12); (1) bellied (Kamiński and Raś 2011: 650).

18. Intercoxal process of prosternum: (0) not widened at the apex; (1) strongly widened at the apex.

**Figures 8–13.**
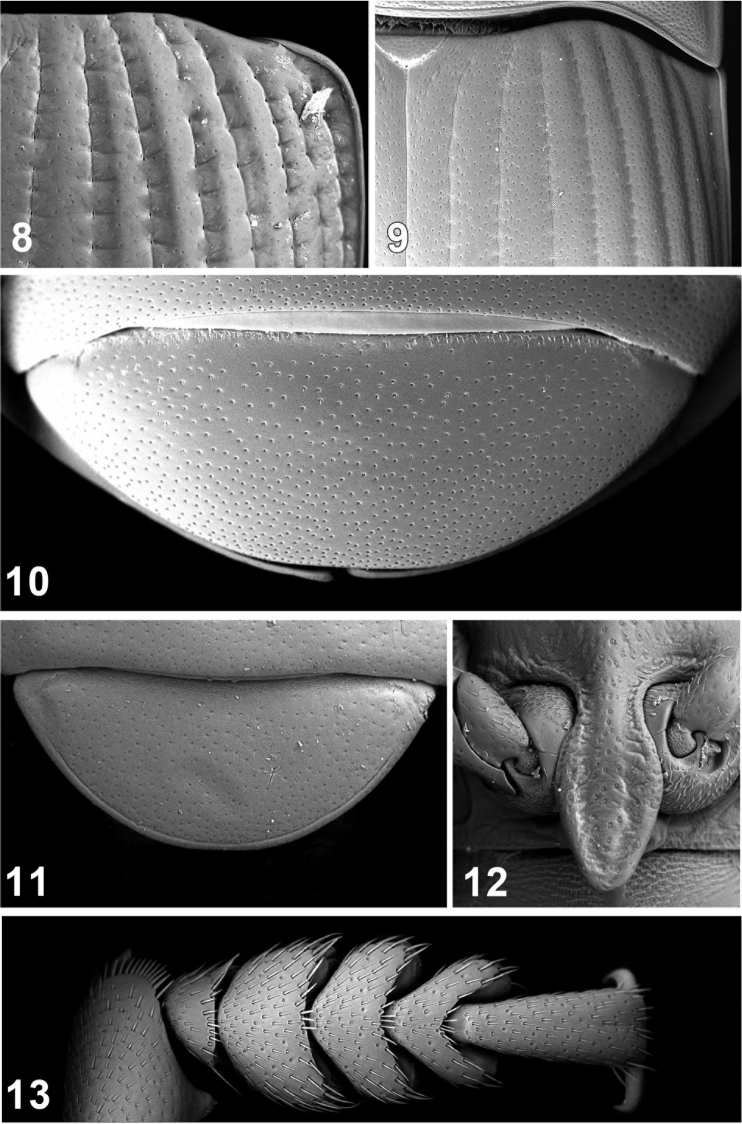
Elytral disc (**8, 9**); 5th abdominal ventrite (**10, 11**), intercoxal process of prosternum (**12**), male protarsi (**13**). *Ectateus crenatus* (**8, 12**), *Monodius convexipennis* (**10**), *Monodius malaisei* (**9**), *Monodius plicicollis* (**13**), *Selinus striatus* (**11**).

**Mesothorax** (characters 19–24)

19. Scutellum: (0) situated at the level of elytra; (1) impressed.

20. Elytral surface: (0) dull; (1) shiny.

21. Elytral intervals with transverse sculpture: (0) no; (1) yes (Fig. 8, 27).

22. Elytral intervals: flat (0); strongly convex (1).

23. Elytral striae: (0) impressed on whole length, with fine punctures (Fig. 9); (1) impressed mainly near conspicuous punctures (Fig. 8).

24. Margins of elytra in basal part: (0) rounded; (1) subparallel (elytral humeri slightly protruding outwards).

**Metathorax** (character 25)

25. Metaventrite: (0) without a coarse longitudinal depression; (1) with a coarse longitudinal depression.

**Abdomen** (characters 26–27)

26. 5^th^ abdominal ventrite: (0) relatively narrow; (1) strongly widened (Fig. 10).

27. 5^th^ abdominal ventrite: (0) unbordered (Fig. 10); (1) bordered (Fig. 11).

**Legs** (character 28–31)

28. Male protarsi widened: (0) no; (1) yes (Fig. 13).

29. Female protarsi widened: (0) no; (1) yes.

30. Male profemora (0) relatively wide (length/width = 3.2-3.6); (1) relatively slender (length/width = 4.0-5.6).

31. Denticle at the apex of the inner face of male mesotibia: (0) small, sometimes absent; (1) large (Fig. 7).

**Male and female genitalia** (character 32–39)

32. Penis wide: (0) no (Figs 20–21); (1) yes, at least 4 times wider than clavae (Figs 14–19).

**Figures 14–21.**
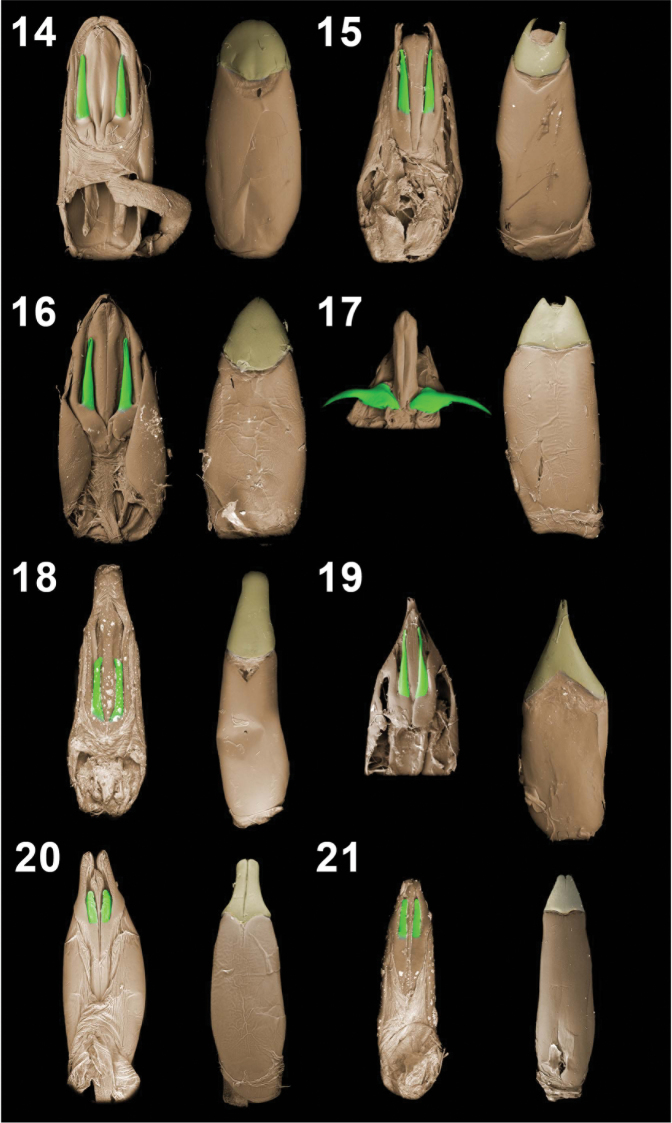
Aedeagal tegmina (dorsal and vental views). *Monodius gravis* (**14**), *Monodius plicicollis* (**15**), *Monodius convexipennis* (**16**), *Monodius malaisei* (**17**), *Monodius lamottei* (**18**), *Monodius laevistriatus* (**19**), *Ectateus calcaripes* (**20**), *Selinus striatus* (**21**).

33. Clavae: (0) straight (Figs 14–19, 21); (1) curved, hook-shaped (Fig. 20).

34. Clavae: (0) short, their length less than half of the length of parameres; long, their length more than half of the length of parameres (1).

35. Parameres strongly extended apically: (0) no; (1) yes (Fig. 18).

36. Parameres narrowest in the half of their length (0) no; (1) yes (Fig. 20).

37. Apex of parameres: (0) not fused (Fig. 20); (1) fused, not emarginated at apex (Figs 14, 16); (2) fused, emarginated at apex (Figs 15, 17).

38. Bursa copulatrix: (0) without additional sacs; (1) with 2 additional sacs (Fig. 23).

**Figures 22–24.**
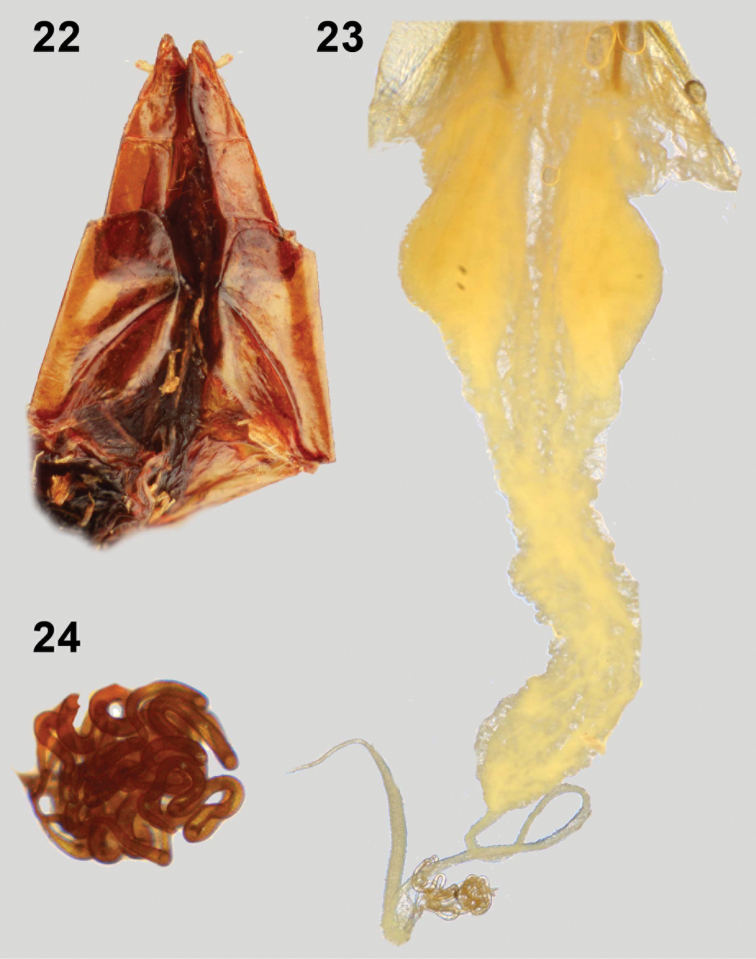
Female genitalia. Ovipositor of *Monodius plicicollis* (**22**); bursa copultrix of *Monodius medius* (**23**), spermatheca of *Monodius gravis* (**24**).

39. Paraproct longer than coxites: (0) no (Fig. 22); (1) yes.

**Other** (character 40)

40. Body size: (0) more than 10.0 mm; (1) less than 9.0 mm.

**Figure 25.**
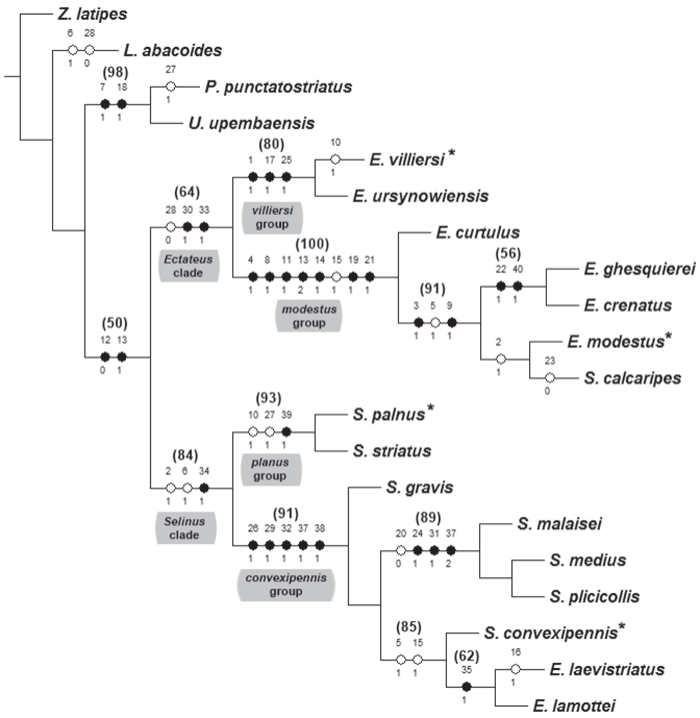
Phylogeny of the species of *Ectateus* and *Selinus* *sensu* Iwan 2002a. Most parsimonious tree (L=57, CI=74, RI=90). Black circles represent single, non-homoplasious character state transformations, and white circles represent multiple, homoplasious character state transformations.The numbers above and below each circle correspond to character numbers and states, respectively. Additional numbers displayed at the top of branches represent Jackknife values (support below 50 was not illustrated). * – type species.

**Phylogenetic analysis.** The cladistic analysis yield a single most parsimonious cladogram (Fig. 25) with a length of 57 steps, a consistency index (CI) of 74 and a retention index (RI) of 90. According to the obtained cladogram the genera *Ectateus* and *Selinus*, in their current interpretations, are paraphyletic (Fig. 25). The *Ectateus* clade is supported by two synapomorphies: male protibia slender (length/width = 4.0-5.6) (char. 30:1) and clavae curved, hook-shaped (char. 33:1). Also one homoplasy was recovered for this clade – male protarsi relatively narrow (char. 28:0). The *Selinus* clade is supported by single synapomorphy – clavae long, their length more than half of the length of parameres (char. 34:1) – and two homoplasies: antennomeres from 7 to 11 elongated (their length greater than the width), anterior tentorial pit deep, clearly visible (char. 2:1, 6:1). The monophyly of the *Ectateus*+*Selinus* clade was supported during the analysis by the following two synapomorphies: ratio of prothorax width (tw) and pronotal disc height (dh) greater than 5 (char. 12: 0) and apophyseal depressions on pronotal disc present (char. 13:1).

Two main species groups were recovered within the *Ectateus* clade – *modestus* group and *villiersi* group. The branch support reported for these groups was relatively high (Fig. 25). The phylogenetic relationships within the *modestus* group were supported unequally. Relatively low Jackknife values were reported within the clade composed of *Ectateus modestus*, *Selinus calcaripes*, *Ectateus ghesquierei* and *Ectateus crenatus*.

According to the results of a cladistic analysis the *modestus* group is characterized by following synapomorphies: fronto-clypeal suture coarse, clearly visible (char. 4:1), anterior pronotal angles curved outwards (char. 8:1), pronotal margins strongly erected upwards (char. 11:1), apophyseal depressions rounded (char. 13:2), pronotal base narrower than elytral base (char. 14:1), scutellum impressed (char. 19:1) and elytral intervals with transverse sculpture (char. 21:1). Additionally, one homoplasy was recovered: posterior pronotal angles strongly protruding towards elytra (char. 15:1).

Despite the fact that the species aggregated in the *modestus* group (Fig. 25) are homogeneous in their morphology the cladistic analysis revealed some species groups. According to the results *Ectateus curtulus* is a sister taxon to all other *modestus* group species. This relationship is supported highly supported (Jackknife support = 91; char. 3:1, 6:1, 9:1). Unfortunately, *Ectateus curtulus* is known only form a single specimen (holotype, female), therefore the above mentioned phylogenetic hypothesis should be reconsidered once the male specimen will be found.

The four remaining species occurred in two separate clades (Fig. 25). The first clade which consists of *Ectateus ghesquierei* and *Ectateus crenatus* is defined by the following synapomorphies: convex elytral intervals (char. 22:1) and a small body size (char. 40:1). The other clade composed of *Ectateus modestus* and *Ectateus calcaripes* comb. n. is only supported by a single homoplasy - antennomeres from 7 to 11 elongated (char. 2:1). However, these two species are very similar in general morphology – the females are almost impossible to separate or distinguish (Figs 45, 49).

According to the results of a cladistic analysis the *villiersi* group is characterized by following synapomorphies: anntenna robust, shorter than pronotum (char. 1:1), intercoxal process of prosternum bellied (char. 17:1) and metaventrite with coarse longitudinal depression (char. 25: 1).

Taking into consideration other significant morphological differences between *modestus* and *villiersi* groups (char. 1, 4, 8, 11, 13–15, 17, 19, 21, 25) it is reasonable to treat them as two separate genera.

Two main highly supported species group were recovered within the *Selinus* clade – *convexipennis* group and *planus* group (Fig. 25). The first group contains the type species (*convexipennis*) of *Monodius* Koch, 1956 (genus synonimized with *Selinus* by Iwan in 2002a).

According to the results of a cladistic analysis the *convexipennis* group is characterized by following synapomorphies: 5^th^ abdominal ventrite strongly widened (char. 26:1), female protarsi widened (char. 29:1), penis wide, at least 4 times wider than clavae (char. 32:1), apex of parameres fused (char. 37:1) and bursa copulatrix with 2 additional sacs (char. 38:1).

*Selinus gravis* occurred as a sister taxon to all other *convexipennis* group species, however this relationship is not highly supported (Fig. 25). The remaining species of the above mentioned group were divided into two separate clades (Fig. 25). The first one which consists of *Selinus malaisei*, *Selinus medius* and *Selinus plicicollis* is defined by the following synapomorphies: margins of elytra in basal part subparallel (elytral humeri slightly protruding outwards) (char. 24:1), denticle at the apex of the inner face of male mesotibia large (char. 31:1), apex of parameres fused and emarginated at apex (char. 37:2). This clade is also supported by a single homoplasy – elytral surface shiny (char. 20:0). The second clade (*Selinus convexipennis*, *Selinus laevistriatus* and *Selinus lamottei*) is defined by two homoplasies: indentation between frons and clypeus on the lateral edge deep (char. 5:1) and posterior pronotal angles strongly protruding towards elytra (char. 15:1).

According to the results of a cladistic analysis the *planus* group is characterized by a following synapomorphy: paraproct longer than coxites (char. 39:1). Additionally, two homoplasies were recovered: pronotum widest at the base (char. 10:1) and 5^th^ abdominal ventrite bordered (char. 27:1).

Because of significant morphological differences between *convexipennis* group and *planus* group, especially the ones concerning the male (char. 32, 37) and female genitalia (char. 38, 39), I propose to consider them as two separate genera.

On the basis of the aforementioned results I propose to classify the analyzed ingroup species in four genera: *Ectateus* (based on *modestus* group), *Monodius* stat. r. (based on *convexipennis* group), *Eleoselinus* gen. n. (based on *villiersi* group) and *Selinus* (based on *planus* group).

A new classification and diagnostic characters of the analyzed ingroups species are presented below.

### Genus Ectateus

Koch, 1956

http://species-id.net/wiki/Ectateus

Ectateus Koch, 1956: 230. – Iwan 2001b: 352, 2002a: 66, 2002b: 265, 2004a: 541, 2004b: 739; Iwan and Banaszkiewicz 2007: 725; Kamiński and Raś 2011: 647; Kamiński 2013a: 85; Raś and Kamiński 2013: 381.

#### Type species.

*Anchophthalmus modestus* Fairmaire, 1887; by original designation.

#### Diagnosis.

The following character combination is unique for *Ectateus* within the whole subtribe Platynotina: (1) fronto-clypeal suture coarse, clearly visible, (2) anterior pronotal angles curved outwards, (3) pronotal margins strongly erected upwards, (4) apophyseal depressions rounded, (5) pronotal base narrower than elytral base, (6) posterior pronotal angles strongly protruding towards elytra, (7) scutellum impressed, (8) elytral intervals with transverse sculpture (9) male protarsi relatively narrow, (10) male protibia slender (length/width = 4.0–5.6) and (11) clavae curved, hook-shaped.

#### Distribution.

*Ectateus* specimens have been collected in the following ecoregions of Central Africa (Cameroon, Central African Republic, Democratic Republic of the Congo, Equatorial Guinea, Gabonese Republic, Republic of Rwanda, Republic of the Congo, South Sudan): Albertine Rift montane forests, Angolan Miombo woodlands, Atlantic Equatorial coastal forests, East Sudanian savanna, Mount Cameroon and Bioko montane forests, Northeastern Congolian lowland forests, Northwestern Congolian lowland forests, Northern Congolian forest-savanna mosaic, Southern Congolian forest-savanna mosaic, Western Congolian forest-savanna mosaic (Fig. 41).

#### Species included (5).

*Ectateus calcaripes* (Gebien, 1904), comb. n., *Ectateus crenatus* (Fairmaire, 1897), *Ectateus curtulus* (Fairmaire, 1893), *Ectateus ghesquierei* Koch, 1956 and *Ectateus modestus* (Fairmaire, 1887).

#### Key to the species of *Ectateus*

**Table:** 

1	Clypeus and genae without depressions. Pronotal margins rounded. Elytral intervals with conspicuous punctures	*Ectateus curtulus*
–	Circular depressions on the lateral sides of clypeus and genae (Fig. 2). Pronotal margins sinusoidal (Fig. 5). Elytral intervals without punctures or punctures very fine (Fig. 8, 27)	2
2	Body size: 7.0–9.0 mm. Antennomeres form 7 to 11 transverse. Elytral striae with deep punctures; intervals convex (Fig. 8)	3
–	Body size: 11.5–14.0 mm. Antennomeres form 7 to 11 elongated. Elytral striae with superficial punctures; intervals flat (Fig. 27)	4
3	Pronotal disc with a longitudinal groove in the middle (Fig. 5). Male protibiae as in Fig. 33; mesofemorae simple	*Ectateus ghesquierei*
–	Pronotal disc without longitudinal groove. Male protibiae as in Fig. 34; mesofemorae with a large denticle on the posterior face (Fig. 35)	*Ectateus crenatus*
4	Intercoxal process protruding towards mesoventrite; peaked at the apex. Male protibiae as in Fig. 31	*Ectateus calcaripes*
–	Intercoxal process not protruding towards mesoventrite; rounded at the apex. Male protibiae as in Fig. 32	*Ectateus modestus*

**Figures 26–30.**
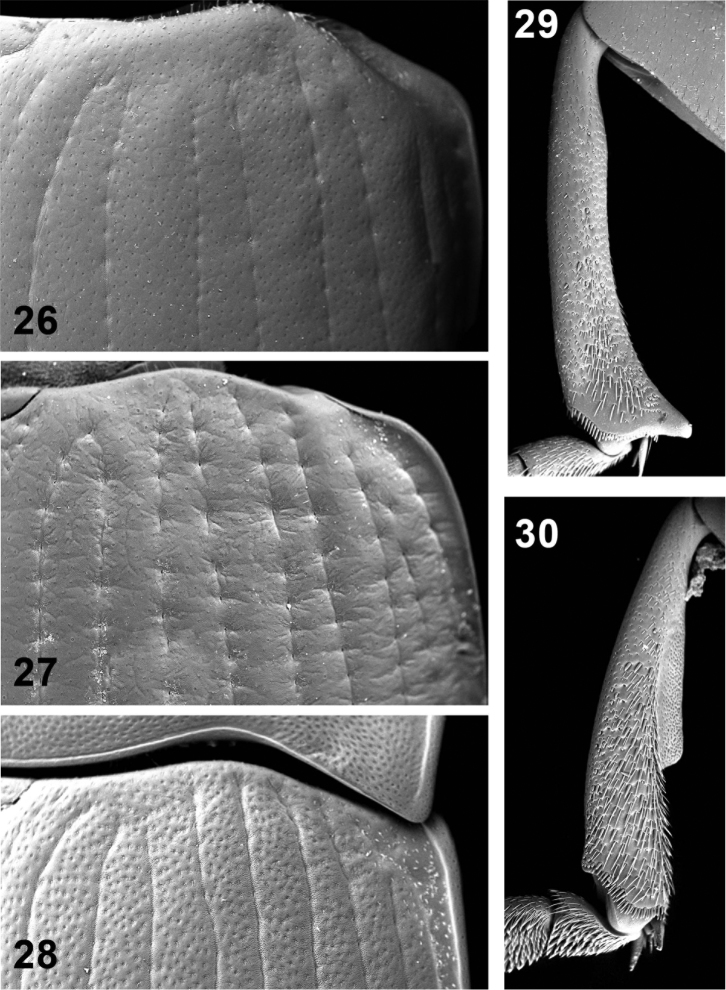
Elytral disc (**26, 27, 28**); male mesotibia (**29**); male protibia (**30**). *Monodius gravis* (**26, 29, 30**), *Ectateus calcaripes* (**27**), *Ectateus lamottei* (**28**).

**Figures 31–36.**
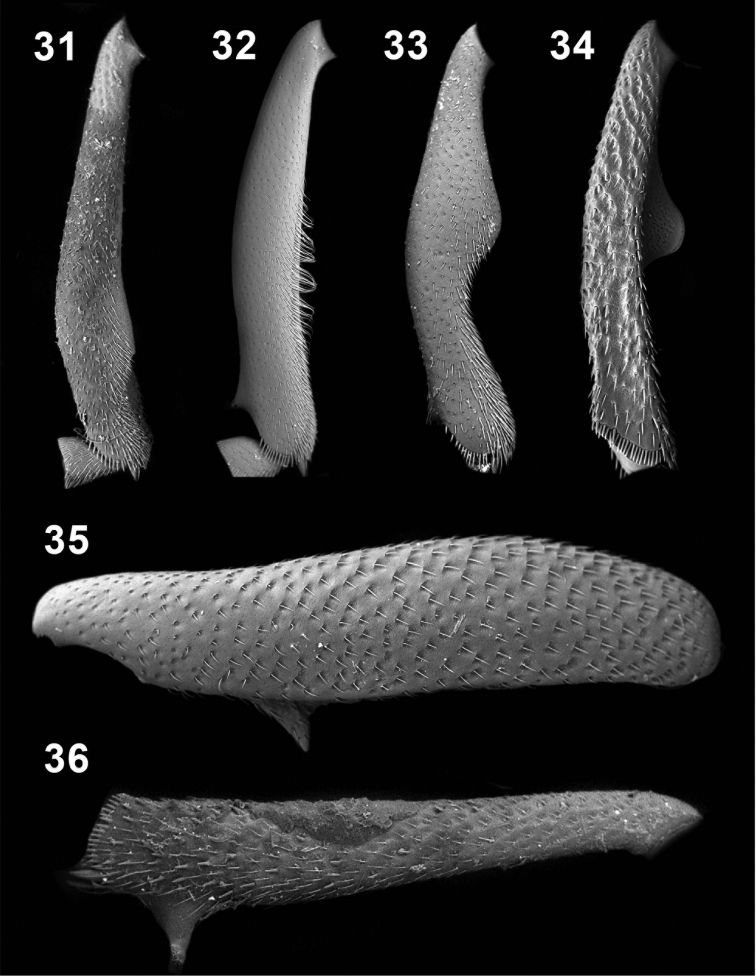
Male protibiae (**31–34**); male mesofemora (**35**); male mesotibia (**36**). *Ectateus calcaripes* (**31**), *Ectateus modestus* (**32**), *Ectateus ghesquierei* (**33**), *Ectateus crenatus* (**34–36**).

### Ectateus calcaripes

(Gebien, 1904) comb. n.

http://species-id.net/wiki/Ectateus_calcaripes

Figs 3 , 20 , 27 , 31 , 41 , 45

Selinus calcaripes Gebien, 1904: 3. – Gebien 1910: 277, 1938: 297; Koch 1956: 238; Kulzer 1963: 425, Iwan 2002b: 265.

#### Notes.

The types of *Selinus calcaripes* seems to be lost. According to the information provided by Iwan (2002b) they should be deposited in Naturhistorisches Museum collection (Basel, Switzerland). Unfortunately, the curators do not confirm this statement. Additionally, during the preparation of my recent scientific project – Phylogeny, biogeography and generic classification of the *Ectateus* generic group (Coleoptera: Tenebrionidae: Platynotina) – I have studied diverse entomological material concerning the subtribe Platynotina from several collections across the World and I did not menaged to locate these specimens.

Based only on the original species descriptions Koch (1956) proposed to consider *Selinus calcaripes* as a synonym of *Ectateus curtulus* (Fairmaire, 1893). Unfortunately, the morphology of the holotype of *Ectateus curtulus* (damaged female – Fig. 47) do not correspond to Gebien’s (1904) description of *Selinus calcaripes* and Koch’s (1956) interpretation of *Ectateus curtulus*. Both publications refer rather to a morphological form that is very closely to *Ectateus modestus* and differers from it mainly by the structure of male protibiae (Figs 31–32). A consistent to the above mentioned descriptions morph was found in the studied material. It was included in the cladistic analysis as *Selinus calcaripes*.

The results of a cladistic analysis confirmed the aforementioned assumption that *Ectateus curtulus* and *Selinus calcaripes* represent two distinct morphological forms (Fig. 25). They can be easily distinguished by the structure of head (char. 3, 5), pronotum (char. 9) and elytra (elytral intervals with conspicuous punctures in *Ectateus curtulus*). Additionally, the results shows that *Selinus calcaripes* is very closely related to *Ectateus modestus* – which is consistent with Gebien’s (1904) description and Koch’s (1956) interpretation.

Taking into consideration the difficulties associated with *Selinus calcaripes* I propose to designate a neotype to clarify the taxonomic status of this species. Additionally, on the basis of the results of a cladistic analysis I propose to treat this taxon as a independent species – not as a synonym of *Ectateus curtulus*.

#### Studied material.

**Neotype** designated here, male (MNHN): “Rep. Centrafric., La Maboke, 2.IX.1967, L. Matile rec.”. **Other material:** male (MNHN): “Musée du Congo, Haut-Uele: Yebo moto, VII-1926, L. Burgeon”, male (MRAC): “Musée du Congo, Haut-Uele: Yebo moto, V-1926, L. Burgeon”, male (MRAC): “Musée du Congo, Bambesa, 15-X-1933, J. V. Leroy”, male and 3 females (MRAC): “Coll. Mus. Tervuren, Oubanghi-Chari: Bangui I/III.1968, ex. coll. Breuning”, male and female (RBINS): “R.I.Sc.Nat. Belg.,I.G. 16.364”, 4 females (MNHN): “Calcaripes, Geb., det. Dr. Kaszab”, male and female (MNHN): “Boukoko, Rep. Centr Afric.”, male and female (MNHN): “La Maboke, Rep. Centr Afric.”, 2 males and female (MNHN): “Lamaboké, 10.XI.1965”, male and female (MNHN): “Lamaboké, 20-21.XI.1966”, male (MNHN): “Lamaboké, 18.XI.1965”, 2 females (HNHM): “Cameroon, Dja Reserve, Ekom, 21-26.XII. 1996, G. Hácz & G. Köszegi”, 3 males (MNB): “Kamerun Buea, 1-7.III.1912, v. Rothkirch S.G.”, female (MNB): “Kamerun, Brea 1000m, 2. - 7. III- 1912; v. Rothkirch S.G.”, female (MNB): “Kamerun, Soppo 25. II. 1912, v. Rothkirch S.G.”, 2 males (MNHN): “Lamaboké, Rep. Centrafric., V-1964, J. Carayon”, female (MNHN): “1968-69, La Maboké, Rép. Centre Afr., P. Teochhi leg.”, male (MNHN): “21.VIII.1969, La Maboké, Rép. Centre Afr., P. Teochhi leg.”, female (RBINS): “Coll. R. I. Sc. N. B., congo belge Beni, crottes d’ éléphants, 18-I-1952 Ch. Verbeke”, female (MNHN): “Muséum Paris, La Maboké, Rép. Centrafric.”, male (MRAC): “Musée du Congo, Région de Sassa, 1895-96, Colmant”, male and female (MNHN): “Cameroun, Dr. G. Nonveiller”, female (MNHN): “Muséum Paris, Congo Moyen, Rég. de M`Baiki, (D Fidao), Pitard 1919”, male (MNHN): “Uganda Prot., Mabira Forest, Chagwe., 3,500-3,800 ft.”, female (MNHN): “Uganda, Nimuli to, Murchison Falls”, male (MNB): “Neu-Kamerun, No. 3360-71, Tessmann S.G.”

#### Redescription.

Habitus as in Fig. 45. Body length = 11.5–14.0 mm. Elytra wider and longer than pronotum (width ratio elytra / pronotum = 1.1–1.2; length ratio elytra / the middle of pronotum = 2.4–2.6).

Dorsal side of head dull, with punctures (the intervals between the punctures are smaller than the diameter of the puncture). Frontoclypeal suture coarse. Clypeal emargination relatively deep (clypeal emargination width / depth ratio = 8.0–8.6). Mentum with median part narrow. Submentum with short base. Maxillary palp not widened (width of maxillary palp / length of 3^rd^ antennomere = 1.0–1.1). Length of antennae greater than pronotal length (ratio antenna / pronotum from tip of anterior pronotal angle to tip of posterior pronotal angle = 1.1–1.2). 3^rd^ antennomere relatively long (length ratio of antennomere 3^rd^ / 2^nd^ = 2.8–3.0).

Pronotal disc transverse (middle of pronotum length / width ratio = 0.4–0.5); dull, with coarse punctures (the intervals between the punctures are smaller than the diameter of the puncture). Anterior pronotal angles sharp and protruding outwards. Lateral margins of pronotal disc sinusoidal. Apophyseal and basal depressions on pronotal disc present; apophyseal depressions rounded. Pronotal hypomera dull; without punctures.

Elytra oblong (elytra length / width ratio = 1.1–1.2). Elytral striae with fine punctures; intervals non-convex, with transverse sculpture (Fig. 27). Elytral base slightly rounded. Elytral humeri rounded, not protruding laterad. Wings absent. Scutellum triangular; situated in a depression.

Intercoxal process protruding towards mesoventrite; peaked at the apex. Metaventrite reduced (length ratio cavity of hind coxa / metaventrite between the insertions of mid and hind coxae ca. 2). In both sexes abdominal process without tubercles; relatively narrow (process of 1^st^ abdominal ventrite / process of metaventrite = 2.1–2.2). 5^th^ abdominal ventrite without bordering; punctures fine (the intervals between the punctures are greater than the 2 diameters of the puncture).

Male legs. Protarsi slightly narrow. Protibiae as in Fig. 31. Mesotibiae and mesofemorae with large denticle. Metafemorae with an hair fringe. Female legs simple.

Male genitalia. Parameres narrowest in the half of their length; length equal to the 0.2 of the rest of aedeagal tegmen (Fig. 20). Clavae hook-shaped (Fig. 20). Female genitalia. Paraproct equal to coxites. Bursa copulatrix with a sclerite in the distal part. Spermatheca with narrow ducts.

#### Distribution.

This species has been collected in the following ecoregions of Central Africa (Cameroon, Central African Republic, Democratic Republic of the Congo, South Sudan): Atlantic Equatorial coastal forests, East Sudanian savanna, Mount Cameroon and Bioko montane forests, Northeastern Congolian lowland forests, Northwestern Congolian lowland forests (Fig. 41).

### Ectateus crenatus

(Fairmaire, 1897)

http://species-id.net/wiki/Ectateus_crenatus

Figs 2 , 8 , 12 , 34–36 , 41 , 46

Selinus crenatus Fairmaire, 1897: 121. – Gebien 1910: 277, 1938: 297.
Ectateus crenatus (Fairmaire, 1897). – Koch 1956: 235, Iwan 2002b: 265; Iwan and Banaszkiewicz 2007: 728.
Selinus monardi Kaszab, 1951: 2 (syn. nov.)
Ectateus latipennis Koch, 1956: 234 (syn. nov.). – Iwan 2002a: 67, 2002b: 266.

#### Notes.

While describing *Ectateus latipennis*, Koch has noted that types of *Ectateus crenatus* were unknown to him. The characters used by Koch to separate those two species (body size, pronotum structure) were based only on the Fairmaire (1897) description. During the examination of available material I have not found any consistent morphological characters to separate those two species. Therefore, I propose to consider *Ectateus latipennis* as a synonim of *Ectateus crenatus*.

The examination of the type material representing *Selinus monardi* resulted in similar conclusions – there are no consistent morphological characters to separate it from *Ectateus crenatus*.

#### Studied material.

Three specimens with “type” labels are available. Fairmaire (1897) do not specify the number of individuals on which he described this species. Lectotype designation is needed to fix the taxonomic status of the genus and the species. **Lectotype** designated here, male (MNHN): “TYPE”, “Museum Paris, 1906, Coll. L. Fairmaire”, “*Selinus crenatus*”; **Paralectotypes**, male (MNHN), female (MNHN): “TYPE”, “Museum Paris, 1906, Coll. L. Fairmaire”, “*Selinus crenatus* Farim 1896, Congo”, female (MNHN): “*Selinus crenatus* Fm n. sp.”, “TYPE”, “Museum Paris, 1906, Coll. L. Fairmaire”, “Congo”. **Other material:** male (MRAC): “Musée du Congo, Barumbu - VIII-1925, (J. Ghesquière), S.A.R. Prince Léopold” (Holotype of *Ectateus latipennis* Koch, 1956), female (RBINS): “Ibembo, Itimbiri” (Allotype of *Ectateus latipennis* Koch, 1956), male (MNHN): “Kamerun, Joko” (Holotype of *Selinus monardi* Kaszab, 1951), male and female (MNHN): “Joko, Kamerun” (Paratype and allotype of *Selinus monardi* Kaszab, 1951), male and female (MNHN): “Cameroun, Dr. G. Nonveiller”, male and female (MNHN): “Muséum Paris, Cameroun, B. de Miré”, 3 males and 2 females (MNHN): “Ogodué, Lambaréné, R. Ellenberger 1913”, male (MNHN): “Congo Français, region D’ ouesso, Bassin N`Gokko-Sanga, Dr. J. Gravot 1906”, male (MNHN): “Congo Français, Talagouga Prés N`Jolé, legit R. Ellenberger, E. Haug 1906”, male and female (MNHN): “Ogooué, Sam-Kita”, 3 females (MNHN): “Congo, Ogooué, Sam Kita, R. Ellenberger 1910”, 2 males and 2 females (MNHN): “Gabon, Libreville et env.”, male and female (MNB): “Span. Guinea, Nkolentangan, XI. 07-V.08, G. Teßmann S.G.”, female (MNHN): “Benito, Congo Franc.”, female (MNHN): “Gabon, Tholon”, male and female (MNHN): “XII.1970, Mbalmayo, CAMEROUN, Mbarga leg.”, female (MNHN): “Congo belge Centr., Kassai, Edm. Taymans, 1904”, male (MHNG): “CAMEROUN VII.83, Etoubi Assok, à la lumière, F. Notari”, female (MNB): “Neu-Kamerun, No. 3337-52, Tessmann S.G.”, female (MRAC): “Coll. Mus. Congo, Mayumbe: Terr. Tshela, rég. de Mabuba VI-1958, Dr R. Laurent”, male (MRAC): “Mus. Roy. Afr. Centr., Guinée Esp: Bata, Rév. P. Basilio”, male (MNHN): “Kuilu, Fr. Congo., Mocquerys, 1892”, male (RBINS): “Chutes de Samlia, Riv N. Gamie, Mocquereys”.

#### Redescription.

Habitus as in Fig. 46. Body length = 7.0–9.0 mm. Elytra wider and longer than pronotum (width ratio elytra / pronotum = 1.1–1.2; length ratio elytra / the middle of pronotum = 2.4–2.6).

Dorsal side of head shiny, with punctures (the intervals between the punctures are smaller than the diameter of the puncture). Frontoclypeal suture coarse. Clypeal emargination relatively deep (clypeal emargination width / depth ratio = 5.7–6.5). Mentum with median part narrow. Submentum with short base. Maxillary palp not widened (width of maxillary palp / length of 3^rd^ antennomere = 1.0–1.2). Length of antennae greater than pronotal length (ratio antenna / pronotum from tip of anterior pronotal angle to tip of posterior pronotal angle = 1.1–1.3). 3^rd^ antennomere relatively long (length ratio of antennomere 3^rd^ / 2^nd^ = 2.8–3.0).

Pronotal disc transverse (middle of pronotum length / width ratio = 0.5–0.6); shiny, with coarse punctures (the intervals between the punctures are smaller than the diameter of the puncture). Anterior pronotal angles sharp and protruding outwards. Lateral margins of pronotal disc sinusoidal. Apophyseal and basal depressions on pronotal disc present; apophyseal depressions rounded. Pronotal hypomera dull; without punctures.

Elytra oblong (elytra length / width ratio = 1.1–1.2). Elytral striae with coarse punctures (Fig. 8); intervals convex, with transverse sculpture and fine puncturation (Fig. 8). Elytral base slightly rounded. Elytral humeri rounded, not protruding laterad. Wings absent. Scutellum triangular; situated in a depression.

Intercoxal process protruding towards mesoventrite, peaked at the apex, slightly saddle-like. Metaventrite reduced (length ratio cavity of hind coxa / metaventrite between the insertions of mid and hind coxae ca. 2). In both sexes abdominal process without tubercles; relatively narrow (process of 1^st^ abdominal ventrite / process of metaventrite = 2.1–2.2). 5^th^ abdominal ventrite without bordering; punctures fine (the intervals between the punctures are greater than the 2 diameters of the puncture).

Male legs. Protarsi slightly narrow. Protibiae as in Fig. 34. Mesotibiae and mesofemorae with large denticle (Figs 35–36). Metafemorae with an hair fringe. Female legs simple.

Male genitalia. Parameres narrowest in the half of their length; length equal to 0.2 of the rest of aedeagal tegmen. Clavae hook-shaped. Female genitalia. Paraproct equal to coxites. Bursa copulatrix without sclerites. Spermatheca with narrow ducts.

#### Distribution.

This species has been collected in the following ecoregions of Central Africa (Cameroon, Democratic Republic of the Congo, Equatorial Guinea, Gabonese Republic, Republic of the Congo): Atlantic Equatorial coastal forests, Northern Congolian forest-savanna mosaic, Northwestern Congolian lowland forests, Southern Congolian forest-savanna mosaic, Western Congolian forest-savanna mosaic (Fig. 41).

### Ectateus curtulus

(Fairmaire, 1893)

http://species-id.net/wiki/Ectateus_curtulus

Fig. 47

Selinus curtulus Fairmaire, 1893: 143. – Gebien 1910: 277, 1938: 297.
Ectateus curtulus (Fairmaire, 1893). – Koch 1956; Iwan 2002a: 67, 2002b: 265.

#### Studied material.

Holotype (Fig. 47), female (MNHN): „l’Oubanghi”.

#### Redescription.

Habitus as in Fig. 47. Body length ca. 12.5 mm. Elytra wider and longer than pronotum (width ratio elytra / pronotum ca. 1.2; length ratio elytra / the middle of pronotum ca. 2.6).

Dorsal side of head dull, with punctures (the intervals between the punctures are smaller than the diameter of the puncture). Frontoclypeal suture coarse. Clypeal emargination relatively deep (clypeal emargination width / depth ratio ca. 8.1). Mentum with median part wide. Submentum with short base. 3^rd^ antennomere relatively long (length ratio of antennomere 3^rd^ / 2^nd^ ca. 3.0).

Pronotal disc transverse (middle of pronotum length / width ratio ca. 0.5); dull, with coarse punctures (the intervals between the punctures are smaller than the diameter of the puncture). Anterior pronotal angles sharp and protruding outwards. Lateral margins of pronotal disc rounded. Apophyseal and basal depressions on pronotal disc present; apophyseal depressions rounded. Pronotal hypomera dull, without punctures.

Elytra oblong (elytra length / width ratio ca. 1.2). Elytral striae with conspicuous punctures; intervals non-convex, with transverse sculpture and conspicuous punctuation (2 diameters apart). Elytral base slightly rounded. Elytral humeri rounded, not protruding laterad. Wings absent. Scutellum triangular; situated in a depression.

Intercoxal process protruding towards mesoventrite, peaked at the apex, slightly saddle-like. Metaventrite reduced (length ratio cavity of hind coxa / metaventrite between the insertions of mid and hind coxae ca. 2). In both sexes abdominal process without tubercles; relatively narrow (process of 1^st^ abdominal ventrite / process of metaventrite ca. 2.1). 5^th^ abdominal ventrite without bordering; punctures fine (the intervals between the punctures are greater than the 2 diameters of the puncture).

Female legs simple.

Female genitalia. Paraproct equal to coxites. Bursa copulatrix without sclerites. Spermatheca unknown.

#### Distribution.

The only known specimen was collected in the Oubanghi (Central Africa). Because of the general character of the geographical reference it can not be translated into ecoregions.

### Ectateus ghesquierei

Koch, 1956

http://species-id.net/wiki/Ectateus_ghesquierei

Figs 5 , 33 , 41 , 48

Ectateus ghesquierei Koch, 1956: 232. – Iwan 2002a: 67, 2002b: 265.

#### Studied material.

Six syntypes of *Ectateus ghesquierei* are available. Lectotype designation is needed to fix the taxonomic status of the genus and the species. **Lectotype** designated here, (MRAC): “Musée du Congo Belge, Kasai: Kondué, E. Luja”; **Paralectotypes**, 3 females (MRAC): same data, 2 females (MRAC): “Coll. Mus. Congo, Mayidi, 1943, Rév. P. Van Eyen”. **Other material:** female (MNHN): “Voka Congo, X.1977”, male and female (MNHN): “2.11.1963., No 78, sifted litter, leg. Endrõy-Younga” “Soil-Zoological Exp., Congo-Brazzaville, Kindamba, Méya, Louolo river”.

#### Redescription.

Habitus as in Fig. 48. Body length = 8.0–9.0 mm. Elytra wider and longer than pronotum (width ratio elytra / pronotum = 1.1–1.2; length ratio elytra / the middle of pronotum = 2.5–2.6).

Dorsal side of head dull, with punctures (the intervals between the punctures are smaller than the diameter of the puncture). Frontoclypeal suture coarse. Clypeal emargination relatively deep (clypeal emargination width / depth ratio = 9.0–9.3). Mentum with median part narrow. Submentum with short base. Maxillary palp not widened (width of maxillary palp / length of 3^rd^ antennomere = 1.0–1.1). Length of antennae greater than pronotal length (ratio antenna / pronotum from tip of anterior pronotal angle to tip of posterior pronotal angle = 1.1–1.2). 3^rd^ antennomere relatively long (length ratio of antennomere 3^rd^ / 2^nd^ = 2.7–3.0).

Pronotal disc transverse (middle of pronotum length / width ratio = 0.4–0.5); dull, with coarse punctures (the intervals between the punctures are smaller than the diameter of the puncture). Anterior pronotal angles sharp and protruding outwards. Lateral margins of pronotum sinusoidal. Apophyseal and basal depressions on pronotal disc present; apophyseal depressions rounded (Fig. 5). Pronotal hypomera shiny; without punctures.

Elytra oblong (elytra length / width ratio = 1.1–1.3). Elytral striae with conspicuous punctures; intervals convex, with transverse sculpture. Elytral base slightly rounded. Elytral humeri rounded, not protruding laterad. Wings absent. Scutellum triangular, situated in a depression.

Intercoxal process protruding towards mesoventrite, peaked at the apex, slightly saddle-shaped. Metaventrite reduced (length ratio cavity of hind coxa / metaventrite between the insertions of mid and hind coxae ca. 2). In both sexes abdominal process without tubercles; relatively narrow (process of 1^st^ abdominal ventrite / process of metaventrite = 2.0–2.2). 5^th^ abdominal ventrite without bordering; punctures fine (the intervals between the punctures are greater than the 2 diameters of the puncture).

Male legs. Protarsi slightly narrow. Protibiae as in Fig. 33. Mesotibiae with large denticle. Metafemorae without fringle of hairs. Female legs simple.

Male genitalia. Parameres narrowest in the half of their length; length equal to the 0.2 of the rest of aedeagal tegmen. Clavae hook-shaped. Female genitalia. Paraproct equal to coxites. Bursa copulatrix without sclerites. Spermatheca with narrow ducts.

#### Distribution.

This species has been collected in the following ecoregions of Central Africa (Democratic Republic of the Congo, Republic of the Congo): Southern Congolian forest-savanna mosaic, Western Congolian forest-savanna mosaic (Fig. 41).

### Ectateus modestus

(Fairmaire, 1887)

http://species-id.net/wiki/Ectateus_modestus

Figs 32 , 41 , 49

Anchophthalmus modestus Fairmaire, 1887: 282. – Gebien 1910: 278, 1938: 298.
Ectateus modestus (Fairmaire, 1887). – Koch 1956: 241; Ardoin 1965: 964; Iwan 2001b: 359, 2002a: 67, 2002b: 266, 2003: 181, 2004a: 548; Kamiński and Raś 2011: 648.

#### Studied material.

**Lectotype** (designated by Kamiński in Kamiński and Raś 2011), male (MNHN): “Type”, “Muséum Paris, 1906, Coll. L. Fairmaire”; **Paralectotype**, female (MNHN): “Type”, “Muséum Paris, 1906, Coll. L. Fairmaire”, “*Anchophthalmus modestus* (Fairmaire) 1887 [unreadable]”. **Other material:** male and females (MNHN): “Kangu, Mayombe, Congo Belge Dr. Peregl”, 2 males and 2 females (MNHN): “Sibiti, Congo, XI-1963”, 2 males (MNHN): “Franz. Congo”, male and female (MNHN): “2.11.1963., No 78, sifted litter, leg. Endrõy-Younga” “Soil-Zoological Exp., Congo-Brazzaville, Kindamba, Méya, Louolo river”, 2 females (MRAC): “Musée du Congo, Mayumbe: Luali, -IX-1937, Dr. Dartevelle”, female (MRAC): “Coll. Mus. Congo, Mayidi, 1942, Rév. P. Van Eyen”, male (MNHN): “Muséum Paris, Loango, Rochut & Perraudin, 161-96”, male (JFCS):“Luvulu, Kouilau, Congo”, female (JFCS): “Luvulu, Kipanga, Kouilau”, 3 females (JFCS): “Congo, Luvulu, Kipanga, Kouilau”, male (MNHN): “9.XI.1974, Voka près Boko, Rép. Pop. CONGO, Fr. Giov. Onore”, male (MNHN): “10-15.XI.1974, Voka près Boko, Rép. Pop. CONGO, Fr. Giov. Onore”, female (MNHN): “X.1976, Voka près Boko, Rép. Pop. CONGO, Fr. Giov. Onore”, male (RBINS): “Kuimba-Diambo, 15-XI-25, A. Collart”, male (RBINS): “I.R.Sc.N.B. I.G. 25.041, Coll. & det. J. Delève”, 2 females (MRAC): “Coll. Mus. Congo, Mayumbe: T. Kipanzu, de, Singa à Mbomba V/VI-58, Dr R. Laurent”, female (MNHN): label unreadable, male (MRAC): “Coll. Mus. Tervuren, Angola: Benguela, ex. coll. Breuning”, male (SMNS): “Kongo, Voka, XI. 1974, W. Heinkel”, male (MNHN): “Tschiloengo, Congo”, male (RBINS): “R. I. Sc. Nat. Belg., I.G. 16.364”.

#### Redescription.

Habitus as in Fig. 49. Body length = 12.0–14.0 mm. Elytra wider and longer than pronotum (width ratio elytra / pronotum = 1.1–1.2; length ratio elytra / the middle of pronotum = 2.4–2.6).

Dorsal side of head dull, with punctures (the intervals between the punctures are smaller than the diameter of the puncture). Frontoclypeal suture coarse. Clypeal emargination relatively deep (clypeal emargination width / depth ratio = 8.0–8.4). Mentum with median part narrow. Submentum with short base. Maxillary palp not widened (width of maxillary palp / length of 3^rd^ antennomere = 1.0–1.1). Length of antennae greater than pronotal length (ratio antenna / pronotum from tip of anterior pronotal angle to tip of posterior pronotal angle = 1.2–1.3). 3^rd^ antennomere relatively long (length ratio of antennomere 3^rd^ / 2^nd^ = 2.7–3.0).

Pronotal disc transverse (middle of pronotum length / width ratio = 0.4–0.5); dull, with coarse punctures (the intervals between the punctures are smaller than the diameter of the puncture). Anterior pronotal angles sharp and protruding outwards. Lateral margins of pronotum sinusoidal. Apophyseal and basal depressions on pronotal disc present; apophyseal depressions rounded. Pronotal hypomera dull; without punctures.

Elytra oblong (elytra length / width ratio = 1.1–1.2). Elytral striae with fine punctures; intervals non-convex, with transverse sculpture. Elytral base slightly rounded. Elytral humeri rounded, not protruding laterad. Wings absent. Scutellum triangular; situated in a depression.

Intercoxal process not protruding towards mesoventrite, rounded at the apex. Metaventrite reduced (length ratio cavity of hind coxa / metaventrite between the insertions of mid and hind coxae ca. 2). In both sexes abdominal process without tubercles; relatively narrow (process of 1^st^ abdominal ventrite / process of metaventrite ca. 2.0. 5^th^ abdominal ventrite without bordering; punctures fine (the intervals between the punctures are greater than the 2 diameters of the puncture).

Male legs. Protarsi slightly narrow. Protibiae as in Fig. 32. Mesotibiae and mesofemorae with large denticle. Metafemorae with an hair fringe. Female legs simple.

Male genitalia. Parameres narrowest in the half of their length; length equal to 0.2 of the rest of aedeagal tegmen. Clavae hook-shaped. Female genitalia. Paraproct equal to coxites. Bursa copulatrix with a sclerite in distal part. Spermatheca with narrow ducts.

#### Distribution.

This species has been collected in the following ecoregions of Central Africa (Angola, Democratic Republic of the Congo, Republic of Rwanda, Republic of the Congo): Albertine Rift montane forests, Angolan Miombo woodlands, Atlantic Equatorial coastal forests, Western Congolian forest-savanna mosaic (Fig. 41).

### Genus Eleoselinus gen. n.

http://zoobank.org/D78C17B9-5607-472D-BE6F-D28B8C33E23F

http://species-id.net/wiki/Eleoselinus

#### Type species.

*Ectateus villiersi* Ardoin, 1965; here designated.

#### Diagnosis.

The presence of the basal depressions on the pronotal disc place *Eleoselinus* near to (Kamiński 2013c): *Anchophthalmops* Koch, 1956, *Anchophthalmus* Gerstaecker, 1854, *Kochogaster* Kamiński & Raś, 2011, *Ectateus*, *Glyptopteryx* Gebien, 1910, *Microselinus* Koch, 1956, *Monodius*, *Nesopatrum* Gebien, 1920, *Phallocentrion* Koch, 1956, *Phymatoplata* Koch, 1956, *Platykochius* Iwan, 2002, *Platymedvedevia* Iwan & Banaszkiewicz, 2007, *Quadrideres* Koch and *Selinus*.

Non-dimorphic legs distinguish *Eleoselinus* from: *Anchophthalmops*, *Anchophthalmus*, *Ectateus*, *Microselinus*, *Monodius*, *Phymatoplata*, *Platykochius*, *Platymedvedevia* and *Selinus*. From *Kochogaster* it can be easily distinguished by a triangular submentum and lack of sclerites in bursa copulatrix (Kamiński and Iwan 2013). Not parallel body sides separates *Eleoselinus* form *Quadrideres* and *Glyptopteryx*. Additionally, from the latter it can be distinguished by flat elytral intervals and slightly sinusoidal base of pronotum (Iwan 2002). Fine hairs covering the body surface, narrow apical segments of maxillary palps and long basal apophyses of aedeagal tegmen separates *Eleoselinus* from *Phallocentrion* (Iwan 2001a).

The following character combination is unique for *Eleoselinus* within the whole subtribe Platynotina: (1) anntenna robust, shorter than pronotum, (2) shallow anterior tentorial pits, (3) presence of apophyseal and basal depressions on pronotal disc, (4) intercoxal process of prosternum bellied, (5) metaventrite with coarse longitudinal depression, (6) 5^th^ abdominal ventrite unbordered, (7) non dimorphic legs and maillary palps, (8) elytral intervals with fine punctures, (9) curved, hook-shaped clavae and (10) longitudinal coxites of ovipositor.

#### Description.

Body length = 10.5–13.0 mm. Elytra wider and longer than pronotum (width ratio elytra / pronotum = 1.1–1.2; length ratio elytra / the middle of pronotum = 2.4–2.9).

Dorsal side of head dull, with fine punctures (the intervals between the punctures are greater than the 2 diameters of the puncture). Frontoclypeal suture fine. Clypeal emargination relatively deep (clypeal emargination width / depth ratio = 5.5–5.7). Mentum with median part wide. Submentum with short base. Maxillary palp not widened (width of maxillary palp / length of 3^rd^ antennomere = 1.0–1.2). Length of antennae slightly greater than pronotal length (ratio antenna / pronotum from tip of anterior pronotal angle to tip of posterior pronotal angle ca. 0.9). 3^rd^ antennomere relatively long (length ratio of antennomere 3^rd^ / 2^nd^ = 3.2–3.5).

Pronotal disc transverse (middle of pronotum length / width ratio = 0.5–0.6); dull, with fine punctures (the intervals between the punctures are greater than the 3 diameters of the puncture). Lateral margins of pronotum narrowing towards apex. Apophyseal and basal depressions on pronotal disc present. Pronotal hypomera dull, without punctures.

Elytra oblong (elytra length / width ratio = 1.1–1.3). Elytral striae with fine punctures (the intervals between the punctures are greater than the 2 diameters of the puncture). Elytral intervals dull, non-convex, without punctures of with very fine punctuation. Elytral base slightly sinusoidal. Elytral humeri rounded, not protruding laterad. Wings absent. Scutellum triangular.

Intercoxal process of prosternum bellied. Metaventrite reduced (length ratio cavity of hind coxa / metaventrite between the insertions of mid and hind coxae ca. 2), with longitudinal depression. In both sexes abdominal process without tubercles, relatively narrow (process of 1^st^ abdominal ventrite / process of metaventrite = 2.1–2.2). 5^th^ abdominal ventrite without bordering; punctures fine (the intervals between the punctures are greater than the 2 diameters of the puncture).

Legs. Protarsi narrow. Other leg segments simple.

Male genitalia. Parameres narrowing towards apex; length equal to the 0.2 of the rest of aedeagal tegmen. Clavae hook-shaped. Female genitalia. Paraproct equal to coxites. Coxites narrow and long. Bursa copulatrix without sclerites.

#### Etymology.

The name is derived from the combination of *Eleo* (prefix indicating the genus *Eleodes* Eschscholtz, 1829 – a poster beetle genus of the Third International Tenebrionoidea Symposium in Tempe, Arizona) and *Selinus*. This genus is named to thank the Steering Committee of the Third International Tenebrionoidea Symposium: Aaron Smith (lead organizer), Rolf Aalbu, Patrice Bouchard, Kojun Kanda, Nico Franz, Warren Steiner and Quentin Wheeler.

#### Distribution.

*Eleoselinus* gen. n. specimens have been collected in the following ecoregion of Central Africa (Republic of the Congo): Western Congolian forest-savanna mosaic (Fig. 42).

#### Species included (2).

*Eleoselinus villiersi* (Ardoin, 1965), comb. n. and *Eleoselinus ursynowiensis* (Kamiński, 2011), comb. n.

#### Key to the species of *Eleoselinus* gen. n.

**Table:** 

1	Pronotal sides evenly narrowing towards apex. Elytral striae impressed on whole length (see Kamiński and Raś 2011: 651). Intercoxal process of prosternum strongly protruding towards mesosternum (see Kamiński and Raś 2011: 650)	*Eleoselinus villiersi*
–	Pronotal sides rounded. Elytral striae impressed only near the punctures (see Kamiński and Raś 2011: 651). Intercoxal process of prosternum slightly protruding towards mesosternum (see Kamiński and Raś 2011: 650)	*Eleoselinus ursynowiensis*

### Eleoselinus villiersi

(Ardoin, 1965) comb. n.

http://species-id.net/wiki/Eleoselinus_villiersi

Fig. 58

Ectateus villiersi Ardoin, 1965: 964. – Iwan 2002b: 266; Iwan and Banaszkiewicz 2007: 729; Kamiński 2011: 648 (in Kamiński and Raś 2011).

#### Studied material.

**Holotype**, male (MNHN): “Brazzaville, Congo, V-1963”, “Muséum Paris, Mission A. Descarpentries et A. Villers, 1963-1964”. **Other specimens**, female (MNHN): “Allotype” same data as holotype, 7 males and 9 females (MNHN): “Juil 1959, Brazzaville, Congo. L. Vincent”, male “Kimpoko, Kongo”, female (MNHN): “Muséum Paris, Congo, Brazzaville, Mission Chari-Tchad, Dr J. Decorse 1904”.

#### Redescription.

Habitus as in Fig. 58. Body length = 10.5–12.0 mm. Elytra wider and longer than pronotum (width ratio elytra / pronotum = 1.1–1.2; length ratio elytra / the middle of pronotum = 2.7–2.9).

Dorsal side of head dull, with fine punctures (the intervals between the punctures are greater than the 4 diameters of the puncture). Frontoclypeal suture fine. Clypeal emargination relatively deep (clypeal emargination width / depth ratio = 5.5–5.7). Mentum with median part wide. Submentum with short base. Maxillary palp not widened (width of maxillary palp / length of 3^rd^ antennomere = 1.0–1.2). Length of antennae slightly greater than pronotal length (ratio antenna / pronotum from tip of anterior pronotal angle to tip of posterior pronotal angle ca. 0.9). 3^rd^ antennomere relatively long (length ratio of antennomere 3^rd^ / 2^nd^ = 3.2–3.5).

Pronotal disc transverse (middle of pronotum length / width ratio = 0.5–0.6), dull, with fine punctures (the intervals between the punctures are greater than the 3 diameters of the puncture). Anterior pronotal angles rounded and slightly protruding towards apex. Lateral margins of pronotal disc narrowing towards apex. Apophyseal and basal depressions on pronotal disc present. Pronotal hypomera dull, without punctures.

Elytra oblong (elytra length / width ratio = 1.1–1.3). Elytral striae with fine punctures (the intervals between the punctures are greater than the 4 diameters of the puncture). Elytral intervals dull, non-convex, without punctures. Elytral base slightly sinusoidal. Elytral humeri rounded, not protruding laterad. Wings absent. Scutellum triangular.

Intercoxal process of prosternum bellied. Metaventrite reduced (length ratio cavity of hind coxa / metaventrite between the insertions of mid and hind coxae ca. 2); with longitudinal depression. In both sexes abdominal process without tubercles; relatively narrow (process of 1^st^ abdominal ventrite / process of metaventrite = 2.1–2.2). 5^th^ abdominal ventrite without bordering; punctures fine (the intervals between the punctures are greater than the 2 diameters of the puncture).

Legs. Protarsi slightly narrow. Legs simple.

Male genitalia. Parameres narrowing towards apex; length equal to the 0.2 of the rest of aedeagal tegmen. Clavae hook-shaped. Female genitalia. Paraproct equal to coxites. Coxites narrow and long. Bursa copulatrix without sclerites.

#### Distribution.

This species has been collected in the following ecoregion Central Africa (Republic of the Congo).

### Eleoselinus ursynowiensis

(Kamiński, 2011) comb. n.

http://species-id.net/wiki/Eleoselinus_ursynowiensis

Fig. 59

Ectateus ursynowiensis Kamiński, 2011: 648 (in Kamiński and Raś 2011).

#### Studied material.

**Holotype**, male (MNHN): “Juil 1959, Brazzaville, Congo. L. Vincent”, male “Kimpoko, Kongo”; **Paratypes**, 10 males and 9 females (MNHN and MIIZ): same data as holotype; female (MNHN): “Muséum Paris, Congo et Oubanghi, Decaux 1896”; male (MNHN): “Muséum Paris, Congo Franc., Env. De Brazzaville, E. Roubaud et A. Weiss, 1907”.

#### Morphological data.

Because the original description (Kamiński and Raś 2011) of this species is relatively recent and consistent with the description style adopted in this study the morphology of this species was not redescribed.

#### Distribution.

This species has been collected in the following ecoregion Central Africa (Republic of the Congo).

### Genus Monodius

Koch, 1956 stat. r.

Monodius Koch, 1956: 181. – Iwan 2001b: 352, 2002a: 100 (syn. with *Selinus*).

#### Type species.

*Selinus convexipennis* Gebien, 1904; by original designation.

#### Diagnosis.

The following character combination is unique for *Monodius* within the whole subtribe Platynotina: (1) antennomeres from 7 to 11 elongated (their length greater than the width), (2) anterior tentorial pit deep, clearly visible, (3) 5^th^ abdominal ventrite strongly widened, (4) female protarsi widened, (5) penis wide, at least 4 times wider than clavae, (6) clavae long, their length more than half of the length of parameres, (7) apex of parameres fused and (8) bursa copulatrix with 2 additional sacs.

#### Distribution.

*Monodius* specimens have been collected in the following ecoregions of West and Central Africa (Burkina Faso, Cameroon, Federal Republic of Nigeria, Ivory Coast, Republic of Benin, Republic of Ghana, Republic of Liberia, Republic of Niger, Sierra Leone, Togolese Republic): Cross-Sanaga-Bioko coastal forests, Atlantic Equatorial coastal forests, Central African mangroves, Eastern Guinean forests, Guinean forest-savanna mosaic, Mount Cameroon and Bioko montane forests, Northern Congolian forest-savanna mosaic, Northwestern Congolian lowland forests, West Sudanian savanna, Western Guinean lowland forests (Figs 42–43).

#### Species included (7).

*Monodius convexipennis* (Gebien, 1904), *Monodius gravis* Koch, 1956, *Monodius laevistriatus* (Fairmaire, 1897), comb. n., *Monodius lamottei* (Gridelli, 1954), comb. n., *Monodius malaisei* Koch, 1956, *Monodius medius* (Fairmaire, 1897), *Monodius plicicollis* (Fairmaire, 1897), comb. n.

#### Key to the species of *Monodius*

**Table:** 

1	Elytral surface dull. Margins of elytra in the basal part subparallel (elytral humeri slightly protruding outwards). Denticle at the apex of the inner face of male mesotibia large (Fig. 7). Apex of parameres emarginated at the apex (Figs 15, 17)	2
–	Elytral surface shiny. Margins of elytra in the basal part rounded. Denticle at the apex of the inner face of male mesotibia small or absent (e.g. Fig. 29). Apex of parameres connected (Figs 14, 16)	4
2	Pronotal apophyseal depressions coarse. Male mesofemorae with a denticle (similar to the one in *Ectateus laevistriatus*, Fig. 40). Parameres strongly emarginated at the apex (Fig. 15)	*Monodius plicicollis*
–	Pronotal apophyseal depressions fine. Male mesofemorae wihout denticles. Parameres slightly emarginated at the apex (Fig. 17)	3
3	Male protibiae with median dilatation on the inner face (similar to the one in *Monodius convexipennis*, Fig. 37)	*Monodius medius*
–	Male protibiae almost straight (Fig. 38)	*Monodius malaisei*
4	Body size: 16.0–19.0 mm. Elytral intervals with fine punctures (Fig. 26). Male protibiae as in Fig. 30	*Monodius gravis*
–	Body size: 12.0-14.5 mm. Elytral intervals with conspicuous punctures. Male protibiae as in Fig. 37	5
5	Pronotal disc with two circular depressions in the middle. Aedeagal tegmen as in Fig. 19	*Monodius laevistriatus*
–	Pronotal disc wtihout circular depressions. Aedeagal tegmen as in Fig. 16, 18	6
6	Elytral intervals with conspicuous punctures. Elytral apex as in Fig. 51. Aedeagal tegmen as in Fig. 16	*Monodius convexipennis*
–	Elytral intervals with very coarse punctures (Fig. 28). Elytral apex rounded. Aedeagal tegmen as in Fig. 18	*Monodius lamottei*

**Figures 37–40.**
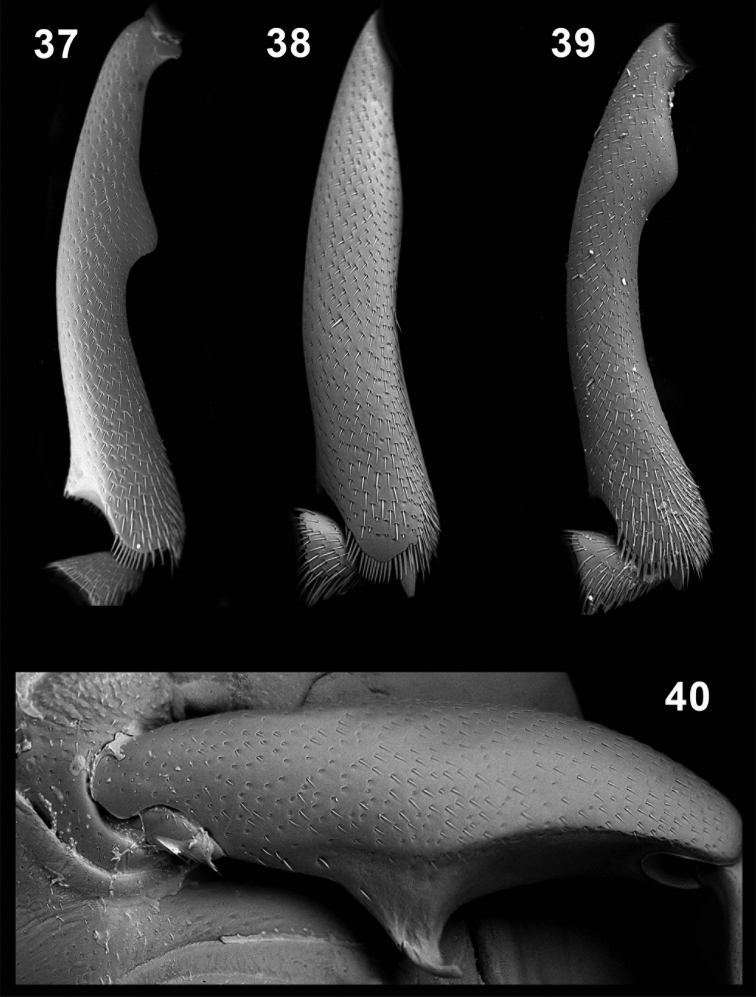
Male protibiae (**37–39**); male mesofemora (**40**). *Monodius convexipennis* (**37**), *Monodius malaisei* (**38**), *Selinus planus* (**39**), *Ectateus laevistriatus* (**40**).

### Monodius convexipennis

(Gebien, 1904)

http://species-id.net/wiki/Monodius_convexipennis

Figs 10 , 16 , 37 , 42 , 50 , 51

Selinus convexipennis Gebien, 1904: 2. – Gebien 1910: 277, 1938: 297; Iwan 2001b: 360, 2002a: 101, 2002b: 302.
Monodius convexipennis (Gebien, 1904). – Koch 1956: 181; Iwan 1990: 430.

#### Studied material.

45 males and 41 females (MIIZ): “Mus. Zool. Polonicum Warszawa 12/45”, “Kamerun, Barombi, Conradt”, “Selinus convexipennis H. Gebien 1939”, 20 males and 12 females (MNHN): “Muséum Paris”, “Kamerun, L. Conradt, 1898-1899”, 3 males and female (MRAC): “Coll. Mus. Tervuren Cameroun, Mt Balmayo (M. Barga), XII 1972, Ex. Coll. Breuning”, female (MRAC): “Coll. Mus. Tervuren, Oubanghi-Chari”, “ex. Coll. Breuning”, 4 females (MNHN): “Muséum Paris, 1952, coll. R. Oberthur”, “Afriq Occid, Johann-Albrechts Höhe, Station Kamerun, L.Conradt, 1896”, male (MNHN): “Muséum Paris, coll. P. Ardoin, 1978”, “Cameroun Yaoundé Vadon!”, 3 males and 4 females (MNB): “Kamerun, John.Albreschtshöhe, 8.11.1898-10.3.1899, leg Conradt”, 6 males and 4 females (MNB): “N.W.Kamerun, Moliwe b. Victoria, 10-20.12.07, Frfr. v. Maltzan G.”, male and 6 females (MNB): “N.-Kamerun, Joh.-Albrechtshöhe, III.96, L.Conradt S. ”, 2 males (MNB): “S.-Kamerun, Bipindi, IX.1898, Zenker S. V.”, 2 females (MNB): “Kamerun, Bascho, Adamatz S.G.”, male and females (MNB): “Kamerun, Nokundange, 16-30 VI 05, G. Leßmann S. G.”, “1908-09.”, male (MNB): “Neu-Kamerun, Dengdeng Station, 26.III.1914, Mildbraed S.G.”, male (MNB): “Kamerun, Duala, Schäfer S.G.”, 3 males (MNHN): “Muséum Paris ex. Coll. P. Ardoin 1978”, “Duala Bothkiych”, 3 males and female (SMNS): “Malimba Pahl 91”.

#### Redescription.

Habitus as in Fig. 50. Body length = 13.0–14.5 mm. Elytra wider and longer than pronotum (width ratio elytra / pronotum = 1.1–1.2; length ratio elytra / the middle of pronotum = 2.4–2.6).

Dorsal side of head dull, with punctures (the intervals between the punctures are smaller than the diameter of the puncture). Frontoclypeal suture fine. Clypeal emargination relatively deep (clypeal emargination width / depth ratio = 7.0–7.5). Mentum with median part wide. Submentum with short base. Maxillary palp not widened (width of maxillary palp / length of 3^rd^ antennomere = 1.1–1.2). Length of antennae greater than pronotal length (ratio antenna / pronotum from tip of anterior pronotal angle to tip of posterior pronotal angle = 1.2–1.3). 3^rd^ antennomere relatively long (length ratio of antennomere 3^rd^ / 2^nd^ = 2.8–2.9).

Pronotal disc transverse (middle of pronotum length / width ratio = 0.5–0.6), dull, with fine punctures (the intervals between the punctures are smaller than the diameter of the puncture). Anterior pronotal angles sharp and strongly protruding towards front. Lateral margins of pronotal disc subparallel at their basal half. Apophyseal and basal depressions on pronotal disc present; apophyseal depressions trapezoidal. Pronotal hypomera dull; without punctures.

Elytra oblong (elytra length / width ratio = 1.1–1.2). Elytral striae with fine punctures, impressed on the whole length. Elytral intervals shiny, non-convex, with conspicuous punctures (the intervals between the punctures are smaller than the diameter of the puncture). Elytral base slightly sinusoidal. Elytral humeri rounded, not protruding laterad. Wings absent. Scutellum rounded.

Intercoxal process slightly protruding towards mesoventrite. Metaventrite reduced (length ratio cavity of hind coxa / metaventrite between the insertions of mid and hind coxae ca. 2). In both sexes abdominal process without tubercles, relatively narrow (process of 1^st^ abdominal ventrite / process of metaventrite = 2.1–2.3). 5^th^ abdominal ventrite without bordering; punctures fine (the intervals between the punctures are greater than the 2 diameters of the puncture).

Male legs. Protarsi slightly widened. Protibiae as in Fig. 37. Mesofemorae with a small denticle at the apex. Metatibiae and Metafemorae with an hair fringe. Female legs. Protarsi slightly widened. Other leg parts simple.

Male genitalia. Parameres narrowing towards apex; length equal to the 0.3 of the rest of aedeagal tegmen (Fig. 16). Clavae straight (Fig. 16). Female genitalia. Paraproct equal to coxites. Bursa copulatrix with two sacs. Spermatheca with narrow ducts.

#### Distribution.

This species has been collected in the following ecoregions of Central Africa (Cameroon): Atlantic Equatorial coastal forests, Central African mangroves, Cross-Sanaga-Bioko coastal forests, Mount Cameroon and Bioko montane forests, Northern Congolian forest-savanna mosaic, Northwestern Congolian lowland forests (Fig. 42).

**Figure 41.**
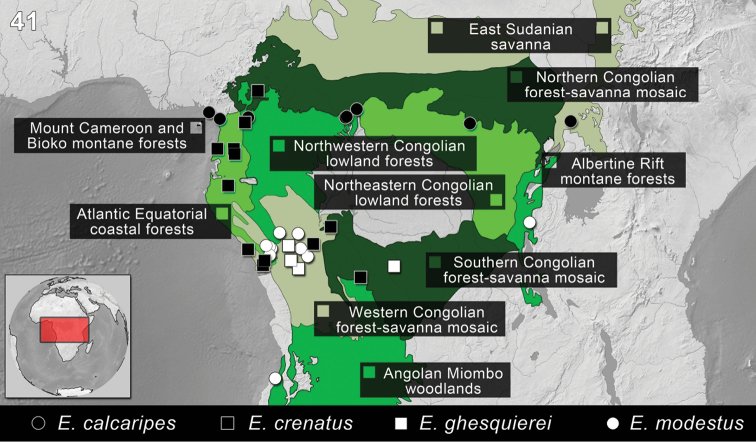
Distribution of the species of *Ectateus* *sensu novum*. The division of Afrotropical Realm into ecoregions was adopted after Olson et al. 2001. Different colors were used to distinguish the adjacent ecoregions.

**Figure 42.**
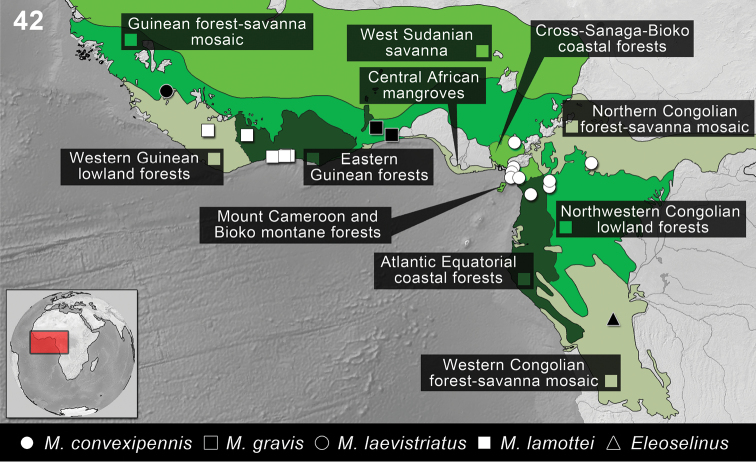
Distribution of the species of *Monodius convexipennis*, *Monodius gravis*, *Monodius laevistriatus*, *Monodius lamottei* and *Eleoselinus* gen. n. The division of Afrotropical Realm into ecoregions was adopted after Olson et al. 2001. Different colors were used to distinguish the adjacent ecoregions.

### Monodius gravis

Koch, 1956

http://species-id.net/wiki/Monodius_gravis

Figs 14 , 24 , 26 , 29 , 30 , 42 , 52

Monodius gravis Koch, 1956: 184.
Selinus gravis (Koch, 1956). – Iwan 2002a: 101, 2002b: 302.

#### Studied material.

Female (MNHN): “Schantung, Tsingtau, Miss. Mus. Steyl”, 3 males and 2 females (MNHN): “Muséum Paris, Bas Dahomey, Torricada E. Poisson 1902”, “Novembre”, female (MNB): “Süd Togo zw. Haho u. Shioftuss Laadschaften Gamme u. Gaohe Smend S.”, 2 females (MIIZ): “Museum Zool. Polonicum, Warszawa, 12/45”, “Selinus n.sp. H. Gebien det. 1939”, “Guinea”, male (MNHN): “Wydah R. P. Ménager”.

#### Redescription.

Habitus as in Fig. 52. Body length = 16.0–19.0 mm. Elytra wider and longer than pronotum (width ratio elytra / pronotum = 1.1–1.2; length ratio elytra / middle of pronotum = 2.2–2.4).

Dorsal side of head dull, with fine punctures (the intervals between the punctures are greater than the 2 diameters of the puncture). Frontoclypeal suture fine. Clypeal emargination relatively deep (clypeal emargination width / depth ratio = 7.1–7.5). Mentum with median part wide. Submentum with short base. Maxillary palp not widened (width of maxillary palp / length of 3^rd^ antennomere = 1.1–1.2). Length of antennae greater than pronotal length (ratio antenna / pronotum from tip of anterior pronotal angle to tip of posterior pronotal angle = 1.2–1.3). 3^rd^ antennomere relatively long (length ratio of antennomere 3^rd^ / 2^nd^ = 2.8–2.9).

Pronotal disc transverse (middle of pronotum length / width ratio = 0.5–0.6), dull, with fine punctures (the intervals between the punctures are greater than the 3 diameters of the puncture). Anterior pronotal angles sharp and strongly protruding toward the front. Lateral margins of pronotal disc rounded. Apophyseal and basal depressions on pronotal disc present; apophyseal depressions trapezoidal. Pronotal hypomera dull; without punctures.

Elytra oblong (elytra length / width ratio = 1.1–1.2). Elytral striae with fine punctures; impressed on the whole length. Elytral intervals shiny, non-convex, with fine punctures (the intervals between the punctures are smaller than the diameter of the puncture). Elytral base slightly sinusoidal. Elytral humeri rounded, not protruding laterad. Wings absent. Scutellum rounded.

Intercoxal process slightly protruding towards mesoventrite. Metaventrite reduced (length ratio cavity of hind coxa / metaventrite between the insertions of mid and hind coxae ca. 2). In both sexes abdominal process without tubercles; relatively narrow (process of 1^st^ abdominal ventrite / process of metaventrite = 2.1–2.3). 5^th^ abdominal ventrite without bordering; punctures fine (the intervals between the punctures are greater than the 2 diameters of the puncture).

Male legs. Protarsi widened. Protibiae as in Fig. 30. Mesofemorae with a small denticle at the apex. Metafemorae with an hair fringe. Female legs. Protarsi slightly widened. Other leg parts simple.

Male genitalia. Parameres narrowing towards apex; length equal to the 0.3 of the rest of aedeagal tegmen (Fig. 14). Clavae straight (Fig. 14). Female genitalia. Paraproct equal to coxites. Bursa copulatrix with two sacs. Spermatheca with narrow ducts (Fig. 24).

#### Distribution.

This species has been collected in the following ecoregions of West Africa (Republic of Benin, Togolese Republic): Guinean forest-savanna mosaic (Fig. 42).

### Monodius laevistriatus

(Fairmaire, 1897) comb. n.

http://species-id.net/wiki/Monodius_laevistriatus

Figs 19 , 40 , 42 , 53

Selinus laevistriatus Fairmaire, 1897: 122. – Gebien 1910: 278, 1921: 21, 1938: 297.
Ectateus laevistriatus (Fairmaire, 1897). – Koch 1956: 237; Ardoin 1969: 143, 1971: 284; Iwan 2002a: 67, 2002b: 265.

#### Studied material.

**Holotype**, female (MNHN): “TYPE”, “Sierra-Leone”, “Selinus laevistriatus Frm [unreadable] Type”, “Muséum Paris, 1906, Coll. L. Fairmaire”. **Other material:** males and 2 female (MNHN): “Fort Camp, 1070m, 26-V-1963”, “Mission ENS-IFAN, aux Monts LOMA, Sierra Leone”, “Muséum Paris, coll. P. Ardoin, 1978”, “*Monodius laevistriatus*, Fairm., P. Ardoin det. 1966”.

#### Redescription.

Habitus as in Fig. 53. Body length = 12.0–14.0 mm. Elytra wider and longer than pronotum (width ratio elytra / pronotum = 1.1–1.2; length ratio elytra / the middle of pronotum = 2.2–2.4).

Dorsal side of head dull, with fine punctures (the intervals between the punctures are smaller than the diameter of the puncture). Frontoclypeal suture fine. Clypeal emargination relatively deep (clypeal emargination width / depth ratio = 7.1–7.5). Mentum with median part wide. Submentum with short base. Maxillary palp not widened (width of maxillary palp / length of 3^rd^ antennomere = 1.1–1.2). Length of antennae greater than pronotal length (ratio antenna / pronotum from tip of anterior pronotal angle to tip of posterior pronotal angle = 1.2–1.3). 3^rd^ antennomere relatively long (length ratio of antennomere 3^rd^ / 2^nd^ = 2.8–2.9).

Pronotal disc transverse (middle of pronotum length / width ratio = 0.5–0.6); dull, with fine punctures (the intervals between the punctures are greater than the 2 diameters of the puncture); with two circular depressions in the middle. Anterior pronotal angles sharp and strongly protruding towards front. Lateral margins of pronotal disc rounded. Apophyseal and basal depressions on pronotal disc present; apophyseal depressions trapezoidal. Pronotal hypomera dull; without punctures.

Elytra oblong (elytra length / width ratio = 1.1–1.2). Elytral striae with fine punctures; impressed on the whole length. Elytral intervals shiny, non-convex, with conspicuous punctures (the intervals between the punctures are smaller than the diameter of the puncture). Elytral base slightly sinusoidal. Elytral humeri rounded, not protruding laterad. Wings absent. Scutellum rounded.

Intercoxal process not protruding towards mesoventrite. Metaventrite reduced (length ratio cavity of hind coxa / metaventrite between the insertions of mid and hind coxae ca. 2). In both sexes abdominal process without tubercles, relatively narrow (process of 1^st^ abdominal ventrite / process of metaventrite = 2.1–2.3). 5^th^ abdominal ventrite without bordering; punctures fine (the intervals between the punctures are greater than the 2 diameters of the puncture).

Male legs. Protarsi slightly widened. Protibiae as in *Monodius convexipennis*. Mesofemorae with a large denticle at the apex, mesotibia with a small denticle at the apex. Metafemorae with an hair fringe. Female legs. Protarsi slightly widened. Other leg parts simple.

Male genitalia. Parameres strongly narrowed toward apex; length equal to the 0.5 of the rest of aedeagal tegmen (Fig. 19). Clavae straight (Fig. 19). Female genitalia. Paraproct equal to coxites. Bursa copulatrix with two sacs. Spermatheca with narrow ducts.

#### Distribution.

This species has been collected in the following ecoregions of West Africa (Sierra Leone): Western Guinean lowland forests (Fig. 42).

### Monodius lamottei

(Gridelli, 1954) comb. n.

http://species-id.net/wiki/Monodius_lamottei

Figs 18 , 28 , 42 , 54

Selinus lamottei Gridelli, 1954: 127.
Ectateus lamottei (Gridelli, 1954). – Ardoin 1963: 222; Iwan 2002b: 266.

#### Studied material.

**Holotype**, male (MNHN): “Muséum Paris, Nimba (Guinée), M. Lamotte II. VI. 42”, “Typus”, “Keoulenta”; **Paratypes**, male and female (MNHN): same data as holotype. **Other material:** 3 females (MNHN): “Muséum Paris, Côte d’Ivoire, Réserve du Banco, R. Paulian & C. Delamare”, 2 males and 2 females (MNHN): “Coll Mus. Tervuren, Côte d’Ivoire: Bingervillie, X.1961, J. Decelle”, “Muséum Paris Coll. P. Ardoin, 1978”, female (MRAC): “Coll Mus. Tervuren, Côte d’Ivoire: Korea, S. de Daloa, J. Decelle, VII / IX.1962”, “*Ectateus laevistriatus*, det. Ardoin 1965”, female (MRAC): “Coll Mus. Tervuren, Côte d’Ivoire: Adlapoté, 80 km. W. Abidjan, J. Decelle, II-1962”, “*Ectateus laevistriatus*, det. Ardoin 1965”, males (TMNH): “Adiopodoumé, B. Côte d’Ivoire, Ledoux”, female (MNHN): “Côte d’Ivoire”, “Muséum Paris Coll. P. Ardoin, 1978”.

#### Redescription.

Habitus as in Fig. 54. Body length = 12.0–14.5 mm. Elytra wider and longer than pronotum (width ratio elytra / pronotum = 1.1–1.2; length ratio elytra / the middle of pronotum = 2.2–2.4).

Dorsal side of head dull, with fine punctures (the intervals between the punctures are smaller than the diameter of the puncture). Frontoclypeal suture fine. Clypeal emargination relatively shallow (clypeal emargination width / depth ratio = 10.0–11.5). Mentum with median part wide. Submentum with short base. Maxillary palp not widened (width of maxillary palp / length of 3^rd^ antennomere = 1.1–1.2). Length of antennae greater than pronotal length (ratio antenna / pronotum from tip of anterior pronotal angle to tip of posterior pronotal angle = 1.2–1.3). 3^rd^ antennomere relatively long (length ratio of antennomere 3^rd^ / 2^nd^ = 2.8–2.9).

Pronotal disc transverse (middle of pronotum length / width ratio = 0.5–0.6), dull, with fine punctures (the intervals between the punctures are smaller than the diameter of the puncture). Anterior pronotal angles sharp and strongly protruding towards front. Lateral margins of pronotal disc rounded. Apophyseal and basal depressions on pronotal disc present; apophyseal depressions trapezoidal. Pronotal hypomera dull; without punctures.

Elytra oblong (elytra length / width ratio = 1.1–1.2). Elytral striae with fine punctures, impressed on the whole length. Elytral intervals shiny, non-convex, with coarse punctures (the intervals between the punctures are smaller than the diameter of the puncture). Elytral base slightly sinusoidal. Elytral humeri rounded, not protruding laterad. Wings absent. Scutellum rounded.

Intercoxal process protruding towards mesoventrite. Metaventrite reduced (length ratio cavity of hind coxa / metaventrite between the insertions of mid and hind coxae ca. 2). In both sexes abdominal process without tubercles, relatively narrow (process of 1^st^ abdominal ventrite / process of metaventrite = 2.1–2.3). 5^th^ abdominal ventrite without bordering; punctures fine (the intervals between the punctures are greater than the 2 diameters of the puncture).

Male legs. Protarsi slightly widened. Protibiae as in *Monodius convexipennis*. Mesotibiae with a small denticle at the apex. Metafemorae with an hair fringe. Female legs. Protarsi slightly widened. Other leg parts simple.

Male genitalia. Parameres extended towards apex; length equal to the 0.5 of the rest of aedeagal tegmen (Fig. 18). Clavae straight (Fig. 18). Female genitalia. Paraproct equal to coxites. Bursa copulatrix with two sacs. Spermatheca with narrow ducts.

#### Distribution.

This species has been collected in the following ecoregions of West Africa (Ivory Coast, Republic of Liberia): Eastern Guinean forests, Western Guinean lowland forests (Fig. 42).

### Monodius malaisei

Koch, 1956

http://species-id.net/wiki/Monodius_malaisei

**Figure 43.**
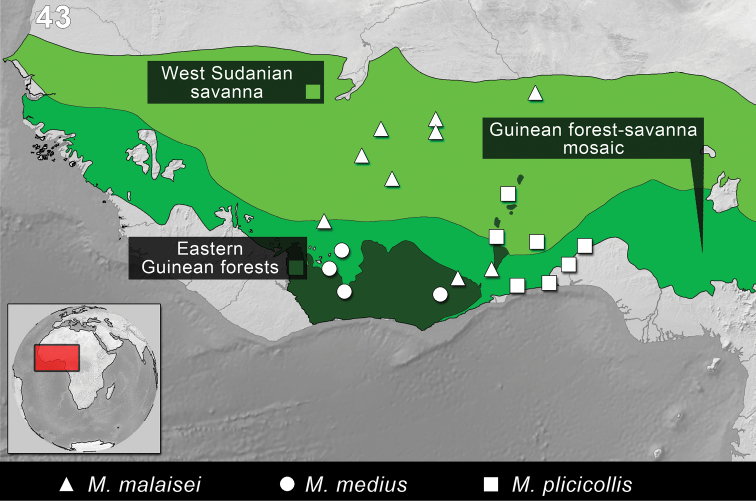
Distribution of the species of *Monodius malaisei*, *Monodius medius* and *Monodius plicicollis*. The division of Afrotropical Realm into ecoregions was adopted after Olson et al. 2001. Different colors were used to distinguish the adjacent ecoregions.

Figs 9 , 17 , 38 , 43 , 55

Monodius malaisei Koch, 1956: 188.
Selinus malaisei (Koch, 1956). – Iwan 2002a: 101, 2002b: 302.

#### Studied material.

Twelve syntypes of *Monodius malaisei malaisei* are available. Lectotype designation is needed to fix the taxonomic status of the species and the subspecies. **Lectotype** designated here, male (TMNH): “Paratype No: 3224, *Monodius malai*-, *sei* Koch”, “Ob. Volta, Pundu, Olsufiew”; **Paralectotypes**, male (TMNH): same data as holotype, except the number referring to paratype (3226, 3227). **Other material:** 12 males and 15 females (MNHN): “Muséum Paris, Haute Volta, Gaoua, H. Labouret 1924”, 2 males and 2 females (ZMAS): “Poundou, Hante, Volta, Afr. Occ. Fr., Oлϲуфъев 927”, male and female (ZMAS): “Ouagadougou, Afrique Occ. Fr. Oлϲуфъев VII. VIII 927”, 3 males and 4 females (MRAC): “Coll. Mus. Tervuren, Haute Volta: Bobo-Dioulasso, 10.V.1964, R. Siffointe”, 2 males (MNHN): “Oyo Yoruba P. François”, “Muséum Paris, Coll. L. Fairmaire”, 2 males (MNHN): “Abétifi, Côte d’Ivoire”, “Muséum Paris”, 2 males (MNHN): “Tortiya, Cte Ivoire, II.59, R. Villemain”, “Muséum Paris”, 2 males (MNB): “Togo, Station Ho, Schroder S.”, male (MNHN): “Muséum Paris Soudan Franc Région Volta Sikasso- Bobo-San A. Chevalier 1900”, “Mai-Juin”, 2 males (MNHN): “Muséum Paris coll. P.Ardoin 1978”, “Monodius Selinus malaisei Koch ssp. nigeriensis Koch P. Ardoin det.1972”, “Niamey-Niger Leg. Loups”, 2 males and 2 females (MNHN): “Muséum Paris, Coll. P. Ardoin, 1978”, “IX.1971, Pabré, Haute Volta, R.P. Fernandez”.

#### Redescription.

Habitus as in Fig. 55. Body length = 13.0-14.0 mm. Elytra wider and longer than pronotum (width ratio elytra / pronotum = 1.2–1.3; length ratio elytra / the middle of pronotum = 2.2–2.4).

Dorsal side of head dull, with fine punctures (the intervals between the punctures are smaller than the diameter of the puncture). Frontoclypeal suture fine. Clypeal emargination relatively shallow (clypeal emargination width / depth ratio = 10.0–11.5). Mentum with median part wide. Submentum with short base. Maxillary palp not widened (width of maxillary palp / length of 3^rd^ antennomere = 1.1–1.2). Length of antennae greater than pronotal length (ratio antenna / pronotum from tip of anterior pronotal angle to tip of posterior pronotal angle = 1.2–1.3). 3^rd^ antennomere relatively long (length ratio of antennomere 3^rd^ / 2^nd^ = 2.8–2.9).

Pronotal disc transverse (middle of pronotum length / width ratio = 0.5–0.6), dull, with fine punctures (the intervals between the punctures are smaller than the diameter of the puncture). Anterior pronotal angles sharp and strongly protruding towards front. Lateral margins of pronotal disc rounded. Apophyseal and basal depressions on pronotal disc present; apophyseal depressions trapezoidal. Pronotal hypomera dull, without punctures.

Elytra oblong (elytra length / width ratio = 1.1–1.2). Elytral striae with fine punctures; impressed on the whole length. Elytral intervals dull, non-convex, with fine punctures (the intervals between the punctures are greater than the 4 diameters of the puncture). Elytral base slightly sinusoidal. Elytral humeri slightly protruding laterad. Wings absent. Scutellum rounded.

Intercoxal process protruding towards mesoventrite. Metaventrite reduced (length ratio cavity of hind coxa / metaventrite between the insertions of mid and hind coxae ca. 2). In both sexes abdominal process without tubercles, relatively narrow (process of 1^st^ abdominal ventrite / process of metaventrite = 2.1–2.3). 5^th^ abdominal ventrite without bordering; punctures fine (the intervals between the punctures are greater than the 2 diameters of the puncture).

Male legs. Protarsi slightly widened. Protibiae as in Fig. 38. Mesotibiae with a large denticle at the apex. Metafemorae with an hair fringe. Female legs. Protarsi slightly widened. Other leg parts simple.

Male genitalia. Parameres extended towards apex; length equal to the 0.2 of the rest of aedeagal tegmen (Fig. 17). Clavae straight (Fig. 17). Female genitalia. Paraproct equal to coxites. Bursa copulatrix with two sacs. Spermatheca with narrow ducts.

#### Distribution.

This species has been collected in the following ecoregions of West Africa (Republic of Ghana, Ivory Coast, Burkina Faso, Federal Republic of Nigeria, Republic of Niger): Eastern Guinean forests, Guinean forest-savanna mosaic, West Sudanian savanna (Fig. 43).

#### Key to the subspecies of *Monodius malaisei*

**Table:** 

1	Male metatibiae curved and slightly dilated on distal half	*Monodius malaisei malaisei* Koch, 1956
–	Male metatibiae straight	*Monodius malaisei nigeriensis* Koch, 1956

### Monodius medius

(Fairmaire, 1897)

http://species-id.net/wiki/Monodius_medius

Figs 7 , 23 , 43 , 56

Selinus medius Fairmaire, 1897: 122. – Gebien 1910: 278, 1938: 297; Iwan 2001b: 360, 2002a: 101, 2002b: 303.
Monodius medius (Fairmaire, 1897). – Koch 1956: 185; Girard 1975: 342.
Selinus angulatipes Gebien, 1921: 21. – Gebien 1938: 297; Koch 1956: 185 (syn); Kulzer 1963: 425.

#### Studied material.

**Holotype**, male (MNHN): “Type”, “Muséum Paris, Paris, Collection Leon Fairmaire, 1906”, “Selinus medius Fm guinea, Acut. det.”. **Other material:** 6 males and 2 females (RBINS): “*Selinus*, *angulatipes*, det. H. Gebien 1913, n. sp.”, “Type”, “Asenté Akem, (Ashanti), Guineé” (Syntypes of *Selinus angulatipes* Gebien, 1921), 11 males and 6 females (MNHN): “Lamto, Pacobo, V. 1968”, “Côte d’Ivoire, CL. Girard Col.”, male (MRAC): “Coll. Mus. Tervuren, Côte d’Ivoire, Kossou 18.2.1975, R. Jocqué”, 2 males (MRAC): “Coll. Mus. Tervuren, Côte d’Ivoire: Bouaké, VII-, 1977, P. M. Elsen”, male and female (MRAC): “Coll. Mus. Tervuren, Togo: Missahoué 650 m., VI.1963, Mme Y. Schach”.

#### Redescription.

Habitus as in Fig. 56. Body length = 12.0–15.0 mm. Elytra wider and longer than pronotum (width ratio elytra / pronotum = 1.2–1.3; length ratio elytra / the middle of pronotum = 2.2–2.4).

Dorsal side of head dull, with fine punctures (the intervals between the punctures are smaller than the diameter of the puncture). Frontoclypeal suture fine. Clypeal emargination relatively shallow (clypeal emargination width / depth ratio = 10.0–11.5). Mentum with median part wide. Submentum with short base. Maxillary palp not widened (width of maxillary palp / length of 3^rd^ antennomere = 1.1–1.2). Length of antennae greater than pronotal length (ratio antenna / pronotum from tip of anterior pronotal angle to tip of posterior pronotal angle = 1.2–1.3). 3^rd^ antennomere relatively long (length ratio of antennomere 3^rd^ / 2^nd^ = 2.8–2.9).

Pronotal disc transverse (middle of pronotum length / width ratio = 0.5–0.6); dull, with fine punctures (the intervals between the punctures are greater than the 2 diameters of the puncture). Anterior pronotal angles sharp and strongly protruding towards front. Lateral margins of pronotal disc rounded. Apophyseal and basal depressions on pronotal disc present; apophyseal depressions trapezoidal. Pronotal hypomera dull, without punctures.

Elytra oblong (elytra length / width ratio = 1.1–1.2). Elytral striae with fine punctures, impressed on the whole length. Elytral intervals dull, non-convex, with fine punctures (the intervals between the punctures are greater than the 4 diameters of the puncture). Elytral base slightly sinusoidal. Elytral humeri slightly protruding laterad. Wings absent. Scutellum rounded.

Intercoxal process not protruding towards mesoventrite. Metaventrite reduced (length ratio cavity of hind coxa / metaventrite between the insertions of mid and hind coxae ca. 2). In both sexes abdominal process without tubercles, relatively narrow (process of 1^st^ abdominal ventrite / process of metaventrite = 2.1–2.3). 5^th^ abdominal ventrite without bordering; punctures fine (the intervals between the punctures are greater than the 3 diameters of the puncture).

Male legs. Protarsi slightly widened. Protibiae as in *Monodius convexipennis*. Mesotibiae with a large denticle at the apex. Metafemorae with an hair fringe. Female legs. Protarsi slightly widened. Other leg parts simple.

Male genitalia. Similar as in *Monodius malaisei*. Female genitalia. Paraproct equal to coxites. Bursa copulatrix with two sacs (Fig. 23). Spermatheca with narrow ducts.

#### Distribution.

This species has been collected in the following ecoregions of West Africa (Republic of Ghana, Ivory Coast): Eastern Guinean forests, Guinean forest-savanna mosaic (Fig. 43).

### Monodius plicicollis

(Fairmaire, 1897) comb. n.

http://species-id.net/wiki/Monodius_plicicollis

Figs 4 , 13 , 15 , 22 , 43 , 57

Selinus plicicollis Fairmaire, 1897: 123. – Gebien 1910: 278, 1938: 297; Koch 1956: 244; Iwan 2002a: 101, 2002b: 303.

#### Studied material.

**Holotype**, male (MNHN): “*Selinus plicicollis*, Fairm, Togo”, “Togoland L. Conradt 1892-1893”, “Muséum Paris, 1906, Coll. L. Fairmaire”; **Paratype**, male (MNHN): same data as holotype. 11 males and 10 females (MRAC): “Coll. Mus. Tervuren Togo: Niamtougou, 21/24-VII-1969, F. Puylaert”, female (MIIZ): “Mus. Zool. Polonicum Warszawa 12/45”, “*Selinus plicicollis* H.Gebien det.1939”, “Dahomey”, male and female (MNHN): “Museum Paris, Moyen-Dahomey, Plateau de Zaguanado, Saison des Drages te. Des Tornades P. Ducorps 1910”, female (MNB): “Togo Bismarckburg, 12-14.IV.93, L. Conradt S.”, male and female (MNHN): “Abboekuta, P. Francois”, “Museum Paris, Coll. Ch. Alluaud, coll. L.Fairmaire 1906”, female (TMNH): “Oyo Yoruba P. François”, “*Selinus plicicollis*”, 7 males and 10 females (MNHN): “Togoland L. Conradt 1892-1893”, “Muséum Paris, 1906, Coll. L. Fairmaire”, 2 females (MNHN): “26.XII.1975, Akoumapé, Togo, J-Cl. Martin”, “Museum Paris, coll. P. Ardoin, 1978”, 6 males and 7 females (MNHN): “Museum Paris Dahomey env. De Porto-Novo, Waterlot 1910”.

#### Redescription.

Habitus as in Fig. 57. Body length = 13.0–18.0 mm. Elytra wider and longer than pronotum (width ratio elytra / pronotum = 1.2–1.3; length ratio elytra / the middle of pronotum = 2.2–2.4).

Dorsal side of head dull, with fine punctures (the intervals between the punctures are smaller than the diameter of the puncture). Frontoclypeal suture fine. Clypeal emargination relatively shallow (clypeal emargination width / depth ratio = 10.0–11.5). Mentum with median part wide. Submentum with short base. Maxillary palp not widened (width of maxillary palp / length of 3^rd^ antennomere = 1.1–1.2). Length of antennae greater than pronotal length (ratio antenna / pronotum from tip of anterior pronotal angle to tip of posterior pronotal angle = 1.2–1.3). 3^rd^ antennomere relatively long (length ratio of antennomere 3^rd^ / 2^nd^ = 2.8–2.9).

Pronotal disc transverse (middle of pronotum length / width ratio = 0.5–0.6); dull, with fine punctures (the intervals between the punctures are greater than the 2 diameters of the puncture). Anterior pronotal angles sharp and strongly protruding towards front. Lateral margins of pronotal disc rounded. Apophyseal and basal depressions on pronotal disc present; apophyseal depressions trapezoidal; very coarse. Pronotal hypomera dull; without punctures.

Elytra oblong (elytra length / width ratio = 1.1–1.2). Elytral striae with fine punctures; impressed on the whole length. Elytral intervals dull, non-convex, with fine punctures (the intervals between the punctures are greater than the 4 diameters of the puncture). Elytral base slightly sinusoidal. Elytral humeri slightly protruding laterad. Wings absent. Scutellum rounded.

Intercoxal process not protruding towards mesoventrite. Metaventrite reduced (length ratio cavity of hind coxa / metaventrite between the insertions of mid and hind coxae ca. 2). In both sexes abdominal process without tubercles; relatively narrow (process of 1^st^ abdominal ventrite / process of metaventrite = 2.1–2.3). 5^th^ abdominal ventrite without bordering; punctures fine (the intervals between the punctures are greater than the 3 diameters of the puncture).

Male legs. Protarsi slightly widened. Protibiae straight. Mesotibiae and mesofemorae with a large denticle at the apex. Metafemorae with an hair fringe. Female legs. Protarsi slightly widened. Other leg parts simple.

Male genitalia. Parameres extended towards apex; length equal to the 0.2 of the rest of aedeagal tegmen (Fig. 15). Clavae straight (Fig. 15). Female genitalia. Paraproct equal to coxites (Fig. 22). Bursa copulatrix with two sacs. Spermatheca with narrow ducts.

#### Distribution.

This species has been collected in the following ecoregions of West Africa (Togolese Republic, Republic of Benin, Federal Republic of Nigeria): Eastern Guinean forests, Guinean forest-savanna mosaic, West Sudanian savanna (Fig. 43).

### Genus Selinus

Mulsant & Rey, 1853

http://species-id.net/wiki/Selinus

Selinus Mulsant & Rey, 1853a: 322. – Lacordaire 1859: 241, Gemminger and de Harold 1870: 1915; Gebien 1910: 277, 1938: 297; Koch 1956: 242; Iwan 2001b: 352, 2002a: 100, 2002b: 302, 2004a: 541, 2004b: 739, 2005: 615; Iwan and Banaszkiewicz 2005: 603, 2007: 725.

#### Type species.

*Opatrum planum* Fabricius, 1792; designated by Gebien (1938).

#### Diagnosis.

The following character combination is unique for *Selinus* within the whole subtribe Platynotina: (1) anterior tentorial pit deep, clearly visible, (2) antennomeres from 7 to 11 elongated (their length greater than the width), (3) pronotum widest at the base, (4) 5^th^ abdominal ventrite bordered, (5) paraproct longer than coxites, (6) clavae long, their length more than half of the length of parameres.

#### Distribution.

**Figure 44.**
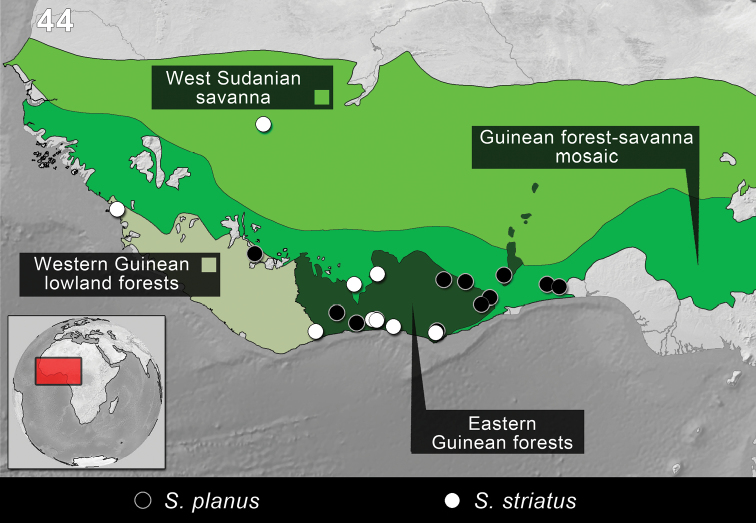
Distribution of the species of *Selinus* *sensu novum*. The division of Afrotropical Realm into ecoregions was adopted after Olson et al. 2001. Different colors were used to distinguish the adjacent ecoregions.

Specimens of this genus have been collected in the following ecoregions of West Africa (Ivory Coast, Republic of Benin, Republic of Ghana, Republic of Guinea, Republic of Mali, Togolese Republic): Eastern Guinean forests, Guinean forest-savanna mosaic, West Sudanian savanna, Western Guinean lowland forests (Fig. 44).

#### Species included (2).

*Selinus planus* (Fabricius, 1792) and *Selinus striatus* (Fabricius, 1794).

#### Key to the species of *Selinus*

**Table:** 

1	Body size: 12.0–14.0 mm. Pronotal sides evenly narrowing towards apex. Pronotal and elytral surface with fine punctures (the intervals between the punctures are greater than the 3 diameters of the puncture). Male protibiae as in Fig. 39	*Selinus planus*
–	Body size: 10.0–11.0 mm. Pronotal sides parallel in their basal half. Pronotal and elytral surface with conspicuous punctures (the intervals between the punctures are smaller than the diameter of the puncture). Male protibiae with very shallow dilatation near the middle	*Selinus striatus*

### Selinus planus

(Fabricius, 1792)

http://species-id.net/wiki/Selinus_planus

Figs 1 , 6 , 39 , 44 , 60

Opatrum planum Fabricius, 1792: 118. – Herbst 1793: 215; Fabricius 1801: 90.
Selinus planus (Fabricius, 1792). – Mulsant and Rey 1853a: 324; Gemminger and de Harold 1870: 1915; Péringuey 1892: 56; Gebien 1906: 211, 1910: 278, 1938: 297; Koch 1956: 251; Iwan 1990: 430, 2001b: 360, 2002a: 101, 2002b: 303; Iwan and Banaszkiewicz 2005: 605.

#### Studied material.

**Paratype**, female (MNHN): “Guinée, coll. R. Oberthür, ex coll. Deyrolle”. **Other material:** 10 males and 9 females (MNHN): “Muséum Paris, Bas Dahomey, Torricada E. Poisson 1902”, “Novembre”, 6 males and 5 females (MNHN): “Muséum Paris Dahomey env. De Porto-Novo, Waterlot 1910”, 3 males (MNHN): “Muséum Paris coll. P. Ardoin 1978”, “15/20.X.1967, Takoradi, Ghana, Cl. Besnard leg.”, “♂”, female (MNHN): “Ashanti”, “Muséum Paris, (Coll. Ch. Alluaud), coll. L.Fairmaire 1906”, 2 males and 3 females (MNB): “Togo Amedzowe”, 2 males and female (MRAC): “Coll. Mus Tervuren, Togo: Missahoué 650 m., VI.1963, Mme Y. Schach”, female (MNHN): “Under log, river bank”, “Akuse, Gold Coast, 23-II-29”, “Museum Paris”, male (MNHN): “Abétifi, Côte d’Ivoire”, “Muséum Paris”, 2 females (MRAC): “Coll. Mus. Tervuren, Ghana: Volta River, 12.VII.1964, G. Marlier”, 2 males (MNHN): “Togo, Palime, Forêt de Klouto, 20-24-IV-74 S. Vit”, female (MRAC): “Coll. Mus. Tervuren, Côte d’Ivoire: Eremankono, S. de Divo, J. Decelle VII-1962”, male and female (MNHN): “Goldküste, Ostertag”, 2 males (MNHN): “Addah.”, “Muséum Paris”, male (MNHN): “Gold Coast”, “Koumassi”, 3 males and female (MNHN): “Dahomey, Athieme, J. M. Renou 1898”, female (BMNH): “Aburi, Gold Coast, W. H. Patterson., 1914-29”, female (MNHN): “Talanzoa”, “Muséum Paris, Nimba (Guinée), M. Lamotte II. VI. 42”.

#### Redescription.

Habitus as in Fig. 60. Body length = 12.0–14.0 mm. Elytra wider and longer than pronotum (width ratio elytra / pronotum = 1.1–1.2; length ratio elytra / the middle of pronotum = 2.7–3.0).

Dorsal side of head dull, with punctures (the intervals between the punctures are smaller than the diameter of the puncture). Frontoclypeal suture fine. Clypeal emargination relatively deep (clypeal emargination width / depth ratio = 4.0–4.5). Mentum with median part wide. Submentum with short base. Maxillary palp not widened (width of maxillary palp / length of 3^rd^ antennomere = 1.0–1.1). Length of antennae greater than pronotal length (ratio antenna / pronotum from tip of anterior pronotal angle to tip of posterior pronotal angle = 1.2–1.3). 3^rd^ antennomere relatively long (length ratio of antennomere 3^rd^ / 2^nd^ = 2.8–3.0).

Pronotal disc transverse (middle of pronotum length / width ratio = 0.4–0.5); dull, with fine punctures (the intervals between the punctures are greater than the 3 diameters of the puncture). Anterior pronotal angles sharp and slightly protruding towards apex. Lateral margins of pronotum narrowing towards apex. Apophyseal and basal depressions on pronotal disc present; apophyseal depressions trapezoidal. Pronotal hypomera dull; without punctures.

Elytra oblong (elytra length / width ratio = 1.1–1.2). Elytral striae with fine punctures (sometimes absent). Elytral intervals shiny, non-convex; with conspicuous punctures (the intervals between the punctures are greater than the 3 diameters of the puncture). Elytral base slightly sinusoidal. Elytral humeri rounded, not protruding laterad. Wings absent. Scutellum triangular.

Intercoxal process protruding towards mesoventrite. Metaventrite reduced (length ratio cavity of hind coxa / metaventrite between the insertions of mid and hind coxae ca. 2). In both sexes abdominal process without tubercles; relatively narrow (process of 1^st^ abdominal ventrite / process of metaventrite = 2.1–2.2). 5^th^ abdominal ventrite with complete bordering; punctures fine (the intervals between the punctures are greater than the 2 diameters of the puncture).

Male legs. Protarsi slightly widened. Protibiae as in Fig. 39. Metafemorae with an hair fringe. Female legs. Protarsi slightly widened. Other leg parts simple.

Male genitalia. Parameres narrowing towards apex; length equal to the 0.2 of the rest of aedeagal tegmen. Clavae straight. Female genitalia. Paraproct longer than coxites. Spermatheca with narrow ducts.

#### Distribution.

This species has been collected in the following ecoregions of West Africa (Ivory Coast, Republic of Benin, Republic of Ghana, Republic of Guinea, Togolese Republic): Eastern Guinean forests, Guinean forest-savanna mosaic, West Sudanian savanna (Fig. 44).

### Selinus striatus

(Fabricius, 1794)

http://species-id.net/wiki/Selinus_striatus

Figs 11 , 21 , 44 , 61

Helops striatus Fabricius, 1794: 440. – Fabricius 1801: 161.
Selinus striatus (Fabricius, 1794). – Gebien 1906: 211, 1910: 278, 1938: 298; Koch 1956: 254; Ardoin 1963: 223, 1969: 143; Iwan 2002b: 303.

#### Studied material.

3 females (MNHN): “Muséum Paris, Côte d’Ivoire, Reserve du Banco, R. Paulian & C. Delamare”, 2 males and female (MNHN): “Muséum Paris (coll. Ch. Alluaud) coll. L. Fairmaire 1906”, “Assinie Côte occid. Afrique Ch. Alluaud 1886”, male and female (MNHN): “Coll Mus. Tervuren, Côte d’Ivoire: Bingervillie, X.1961, J. Decelle”, “Muséum Paris Coll. P. Ardoin, 1978”, female (MRAC): “Cerole de Sassandra, Cote d’Ivoire 4.1962, J.Hamon Orstom Rec.”, “*Selinus striatus* Fab. P. Ardoin det.1963”, “♀”, female (MRAC): “Coll. Mus. Tervuren Côte d’Ivoire: Amanikro, 50 km. N.W. Abengourou J.Decelle V/VI.1961, “Récolté sur cacaoyer”, “Selinus striatus Fab. P.Ardoin det.1965”, male and female (MNHN): “Muséum Paris (coll. Ch.Alluaud) coll. L. Fairmaire 1906”, “Rhobomp, Sierra Leone”, male (MNHN): “Muséum Paris, Iles se Los, Tamara, J. Serand 1913”, male (MHNL): “Dimbokru, Côte d’Ivoire, G. Skibiski, Lyon”, male (MNHN): “Muséum Paris coll. P. Ardoin 1978”, “3.V.1967, Takoradi, Ghana, Cl.Besnard leg.”, “♂”.

#### Redescription.

Habitus as in Fig. 61. Body length = 10.0–11.0 mm. Elytra wider and longer than pronotum (width ratio elytra / pronotum = 1.1–1.2; length ratio elytra / the middle of pronotum = 2.7–2.9).

Dorsal side of head dull, with punctures (the intervals between the punctures are smaller than the diameter of the puncture). Frontoclypeal suture fine. Clypeal emargination relatively deep (clypeal emargination width / depth ratio = 4.0–4.4). Mentum with median part wide. Submentum with short base. Maxillary palp not widened (width of maxillary palp / length of 3^rd^ antennomere = 1.1–1.3). Length of antennae greater than pronotal length (ratio antenna / pronotum from tip of anterior pronotal angle to tip of posterior pronotal angle = 1.2–1.3). 3^rd^ antennomere relatively long (length ratio of antennomere 3^rd^ / 2^nd^ = 2.8–3.0).

Pronotal disc transverse (middle of pronotum length / width ratio = 0.4–0.5); dull, with fine punctures (the intervals between the punctures are smaller than the diameter of the puncture). Anterior pronotal angles sharp and slightly protruding towards apex. Lateral margins of pronotal disc narrowing towards apex. Apophyseal and basal depressions on pronotal disc present; apophyseal depressions trapezoidal. Pronotal hypomera dull; without punctures.

Elytra oblong (elytra length / width ratio = 1.1–1.2). Elytral striae with fine punctures (sometimes absent). Elytral intervals shiny, non-convex, with conspicuous punctures (the intervals between the punctures are smaller than the diameter of the puncture). Elytral base slightly sinusoidal. Elytral humeri rounded, not protruding laterad. Wings absent. Scutellum triangular.

Intercoxal process protruding towards mesoventrite. Metaventrite reduced (length ratio cavity of hind coxa / metaventrite between the insertions of mid and hind coxae ca. 2). In both sexes abdominal process without tubercles; relatively narrow (process of 1^st^ abdominal ventrite / process of metaventrite = 2.1–2.2). 5^th^ abdominal ventrite with complete bordering; punctures fine (the intervals between the punctures are greater than the 2 diameters of the puncture).

Male legs. Protarsi slightly slightly widened. Male protibiae with very shallow dilatation near the mddle. Metafemorae with an hair fringe. Female legs. Protarsi slightly widened. Other leg parts simple.

Male genitalia. Parameres narrowing towards apex; length equal to the 0.2 of the rest of aedeagal tegmen (Fig. 21). Clavae straight (Fig. 21). Female genitalia. Paraproct longer than coxites. Spermatheca and bursa copulatrix as in *Selinus planus*.

#### Distribution.

This species has been collected in the following ecoregions of West Africa (Ivory Coast, Republic of Ghana, Republic of Guinea, Republic of Mali): Eastern Guinean forests, Guinean forest-savanna mosaic, West Sudanian savanna, Western Guinean lowland forests (Fig. 44).

**Figures 45–49.**
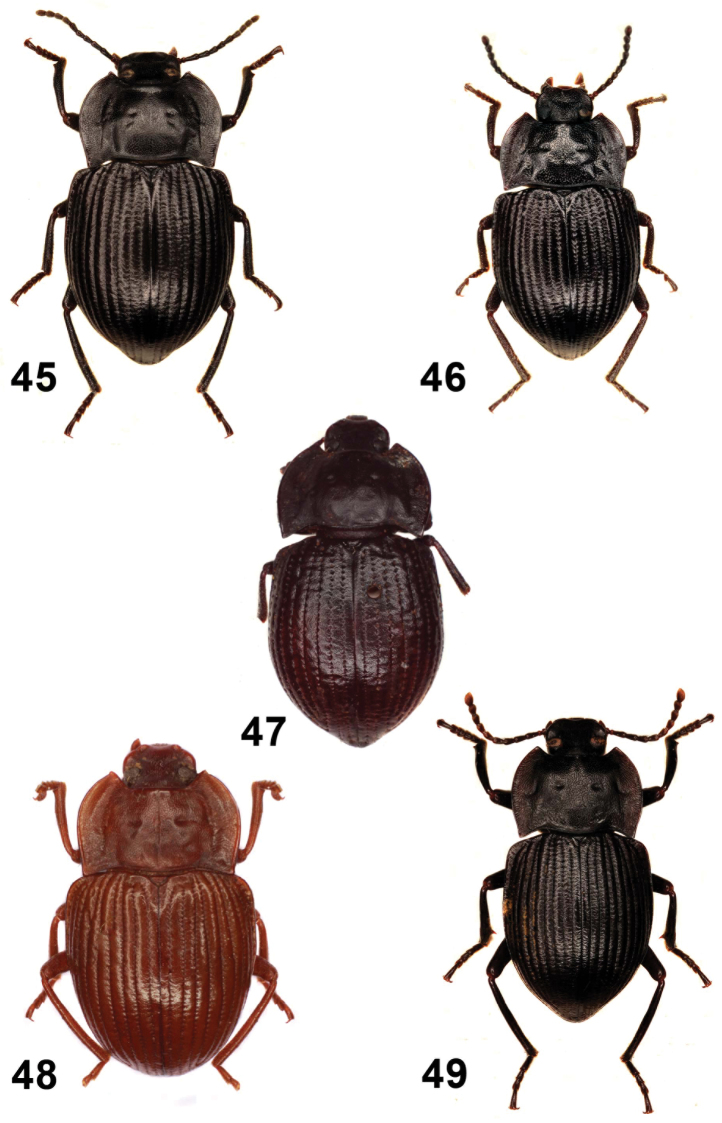
Body habitus: *Ectateus calcaripes* (**45**), *Ectateus crenatus* (**46**), *Ectateus curtulus* (**47**), *Ectateus ghesquierei* (**48**) and *Ectateus modestus* (**49**).

**Figures 50–54.**
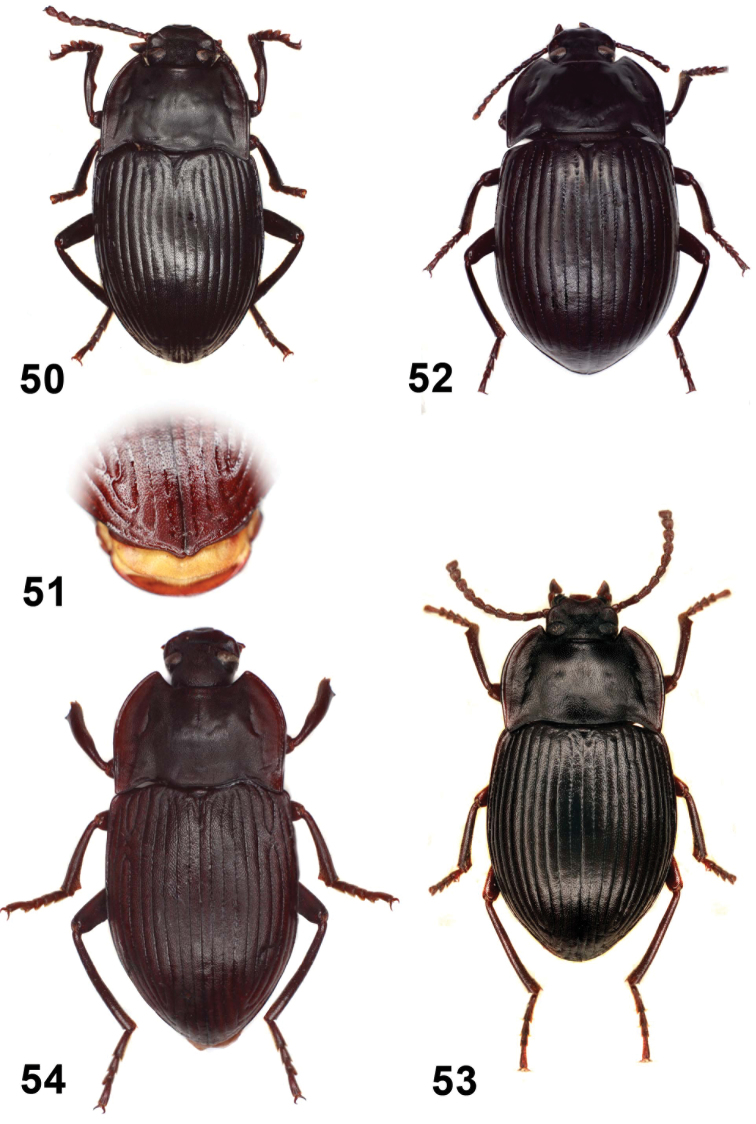
Body habitus: *Monodius convexipennis* (**50**), *Monodius gravis* (**52**), *Monodius laevistriatus* (**53**) and *Monodius lamottei* (**54**). Apex of elytra of *Monodius convexipennis* (**51**).

**Figures 55–59.**
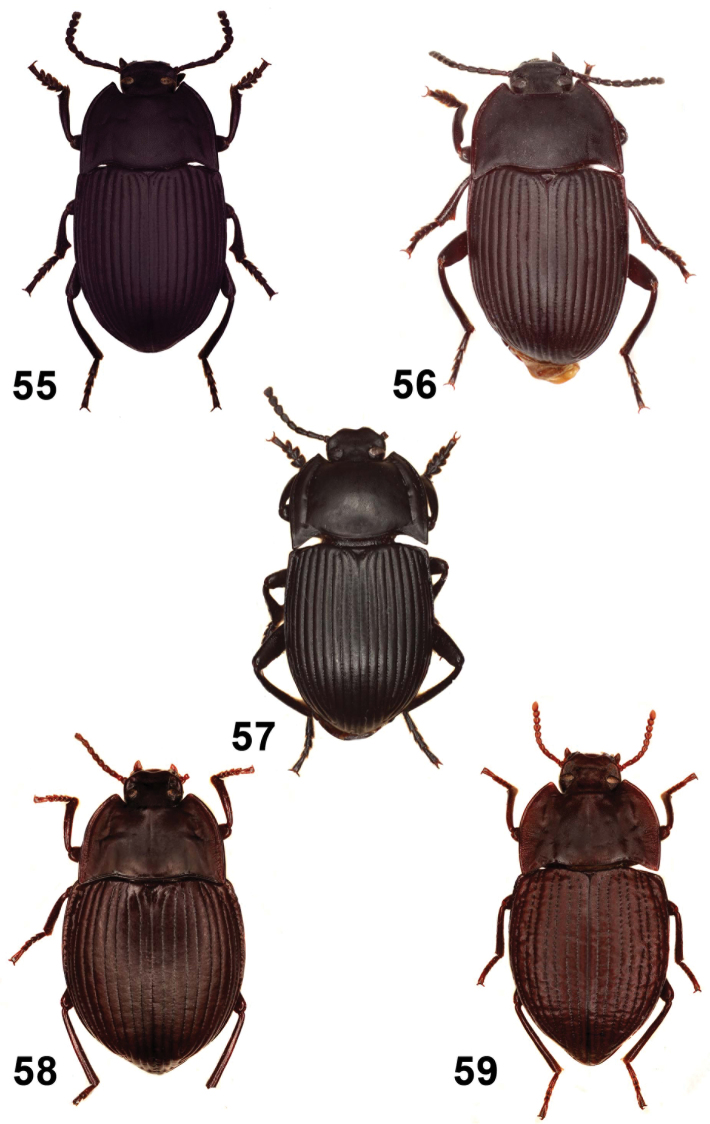
Body habitus: *Monodius malaisei* (**55**), *Monodius medius* (**56**), *Monodius plicicollis* (**57**), *Eleoselinus villiersi* (**58**) and *Eleoselinus ursynowiensis* (**59**).

**Figures 60–61.**
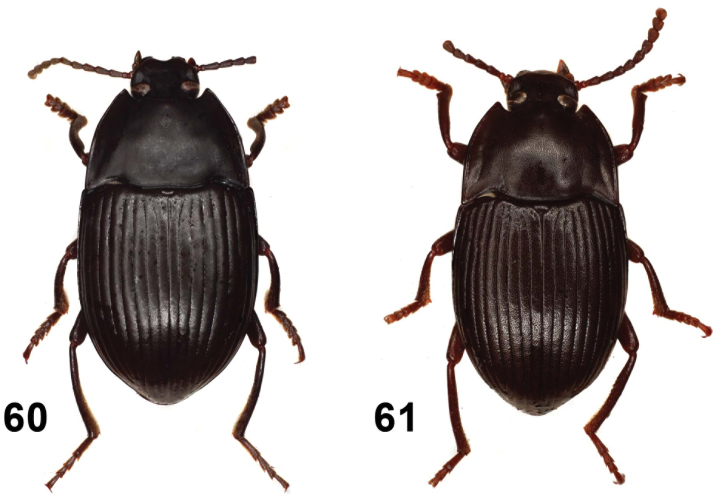
Body habitus: *Selinus planus* (**60**) and *Selinus striatus* (**61**).

## Supplementary Material

XML Treatment for Ectateus

XML Treatment for Ectateus calcaripes

XML Treatment for Ectateus crenatus

XML Treatment for Ectateus curtulus

XML Treatment for Ectateus ghesquierei

XML Treatment for Ectateus modestus

XML Treatment for Eleoselinus

XML Treatment for Eleoselinus villiersi

XML Treatment for Eleoselinus ursynowiensis

XML Treatment for Monodius

XML Treatment for Monodius convexipennis

XML Treatment for Monodius gravis

XML Treatment for Monodius laevistriatus

XML Treatment for Monodius lamottei

XML Treatment for Monodius malaisei

XML Treatment for Monodius medius

XML Treatment for Monodius plicicollis

XML Treatment for Selinus

XML Treatment for Selinus planus

XML Treatment for Selinus striatus

## References

[B1] Ardoin P (1963) La réserve naturelle intégrale du Mont Nimba. III. Coleoptera Tenebrionidae. Mémoires de l’Institut Français d’Afrique Noire 66: 221–267

[B2] Ardoin P (1965) Contribution à la faune du Congo (Brazzaville) (Mission A. Villers et A. Descarpentries). VIII. Coléoptères Ténébrionides. Bulletin de l’Institut Français d’Afrique Noire, Série A, 27: 963–1015

[B3] Ardoin P (1969) Contribution à la connaissance de la Faune Entomologique de la Côte–D’Ivoire (J. Decelle, 1961–1964), Deuxième Partie 37 – Coleoptera, Tenebrionidae. Musée Royal de l’Afrique Centrale, Annales – Serie IN-8° - Sciences Zoologiques 175: 139–285

[B4] Ardoin P (1971) Le massif des Monts Loma (Sierra Leone). XII. Coleoptera Tenebrionidae. Mémoires de l’Institut Français d’Afrique Noire 86: 283–290

[B5] Banaszkiewicz M (2006) Comparative study of female genitalia in Pedinini (sensu Iwan 2004) (Coleoptera: Tenebrionidae: Pedinini), with notes on the classification. Annales Zoologici 56: 59–77

[B6] Bouchard P Lawrence JF Davies A Newton AF (2005) Synoptic classification of the world Tenebrionidae (Insecta: Coleoptera) with a review of family-group names. Annales Zoologici (Warszawa) 55(4): 499–530

[B7] Bouchard P Bousquet Y Davies AE Alonso-Zarazaga MA Lawrence JF Lyal CHC Newton AF Reid CAM Schmitt M Ślipiński SA Smith ABT (2011) Family-group names in Coleoptera (Insecta). ZooKeys 88: 1–972. doi: 10.3897/zookeys.88.807 21594053 10.3897/zookeys.88.807 PMC3088472

[B8] Fabricius JC (1792) Entomologia Systematica emendata et aucta. Secundum classes, ordines, genera, species adjectis synonimis, locis, observationibus, descriptionibus. Hafniae, 1, pars 1, XX+330 pp

[B9] Fabricius JC (1794) Entomologia systematica emendata et aucta adjectis synonymis, loci, observationibus, descriptionibus, Vols. 1–4 Hafniae

[B10] Fabricius JC (1801) Systema Eleutheratorum secundum ordines, genera, species adiectis synonymis, locis, observationibus, descriptionibus. Kiliae, 1, XXIV+506 pp

[B11] Fairmaire L (1887) Coléoptères des Voyages de M. G. Revoil chez les Somalis et dans l’intérieur du Zanguebar. Annales de la Société Entomologique de France 7: 227–368

[B12] Fairmaire L (1893) Coléoptères de l’Oubanghi, recueillis par Crampel. Annales de la Société Entomologique de France 62: 135–146

[B13] Fairmaire L (1897) Coléoptères nouveaux de l’Afrique intertropicale et australe. Annales de la Société Entomologique de France 66: 109–152

[B14] Farris JS (1989) The retention index and the rescaled consistency index. Cladistics 5: 417–419. doi: 10.1111/j.1096-0031.1989.tb00573.x 10.1111/j.1096-0031.1989.tb00573.x 34933481

[B15] Gebien H (1904) Beiträge zur Kenntnis der Insektenfauna von Kamerun. No 28. Verzeichnis der von Professor Dr. Yngve Sjöstedt in Kamerun gesammelten Tenebrioniden. Arkiv Fur Zoologi 2: 1–31

[B16] Gebien H (1906) Über die von Fabricius beschreiben Typen von Tenebrioniden in den Museen von Kopenhagen und Kiel. Deutsche Entomologische Zeitschrift 1: 209–237

[B17] Gebien H (1910) Tenebrionidae I In: W. Junk, S. Schenkling, Coleopterorum Catalogus, Berlin 18: 167–354

[B18] Gebien H (1921) Die Tenebrioniden West–Afrikas. Archiv für Naturgeschichte 86: 1–256

[B19] Gebien H (1938) Katalog der Tenebrioniden. Teil II. Mitteilungen der Muncher Entomologischen Gesellschaft 28: 49–80, 283–428 [370–465].

[B20] Gemminger M Harold E (1870) Catalogus Coleopterorum hucusque descriptorum synonymicus et systematicus. Tenebrionidae, Nilionidae, Pythidae, Melandryidae, Lagriidae, Pedilidae, Anthicidae, Pyrochroidae, Mordellidae, Thipidophoridae, Cantharidae, Oedemeridae 7: 1801–2668

[B21] Girard C (1975) Étude des peuplements de Coléoptères Ténébrionides de la savane de Lamto (Côte D’Ivoire). Annales de la Société Entomologique de France, (Nouvelle série) 2: 335–381

[B22] Goloboff PA Farris J Nixon K (2003) T.N.T. Tree Analysis Using New Technology. http://www.zmuc.dk/public/phylogeny/tnt

[B23] Gridelli E (1954) La réserve naturelle intégrale du mont Nimba. Coléoptères Ténébrionides. Mémoires de l’Institut Français d’Afrique Noire 40: 123–146

[B24] Herbst JFW (1793) Natursystem aller bekannten in– und ausländischen Insecten, als eine Fortsezung der von Büffonschen Naturgeschichte. Der Käfer, Berlin, 5, XV+392 pp, 16 pls

[B25] Hijmans RJ Guarino L Mathur P (2012) DIVA-GIS. Version 7.5. A geographic information system for the analysis of species distribution data. http://www.diva-gis.org

[B26] Iwan D (1990) Platynotini (Coleoptera, Tenebrionidae, Platynotini) of the Natural History Museum in Geneva. Revue suisse de zoologie 97: 427–434

[B27] Iwan D (2000) Oviviparity in tenebrionid beetles of the melanocratoid Platynotina (Coleoptera: Tenebrionidae: Platynotini) from Madagascar with notes on the viviparous beetles. Annales Zoologici 50: 15–25

[B28] Iwan D (2001a) A revision of *Phallocentrion* Koch, 1956 from Africa (Coleoptera: Tenebrionidae: Platynotini). Annales Zoologici 51: 53–63

[B29] Iwan D (2001b) Comparative study of male genitalia in Opatrinae *sensu* Medvedev (1968) (Coleoptera: Tenebrionidae), with notes on the tribal classification. Part I. Annales Zoologici 51: 351–390

[B30] Iwan D (2002a) Generic classification of the tribe Platynotini (Coleoptera: Tenebrionidae), with notes on phylogeny. Annales Zoologici 52: 1–149

[B31] Iwan D (2002b) Catalogue of the World Platynotini (Coleoptera: Tenebrionidae). Genus 13: 219–323

[B32] Iwan D (2003) *Synquadrideres*, new genus of Platynotini from Kenya (Coleoptera: Tenebrionidae). Annales Zoologici 53: 181–187

[B33] Iwan D (2004a) Revision of African *Ectateus* group (Coleoptera: Tenebrionidae: Platynotini). Part I. Introduction and genus *Nesopatrum* Gebien, 1920. Annales Zoologici 54: 541–552

[B34] Iwan D (2004b) A comparative study of male genitalia in Opatrinae *sensu* Medvedev (1968) (Coleoptera: Tenebrionidae), with notes on the reinterpreted tribal classification. Part II. Annales Zoologici 54: 735–765

[B35] Iwan D (2005) Revision of African *Ectateus* group (Coleoptera: Tenebrionidae: Platynotina). Part III. Genus *Platykochius* Iwan, 2002. Annales Zoologici 55: 615–624

[B36] Iwan D Banaszkiewicz M (2005) Revision of African *Ectateus* group (Coleoptera: Tenebrionidae: Platynotina). Part II. Genus *Pseudoselinus* Iwan, 2002. Annales Zoologici 55: 603–613

[B37] Iwan D Banaszkiewicz M (2007) *Platymedvedevia*, a new genus of *Ectateus* group from Tropical Africa (Coleoptera: Tenebrionidae: Platynotina). Annales Zoologici 57: 725–731

[B38] Iwan D Kamiński MJ (2012) Revision of the Malagasy genus *Lechius* Iwan, 1995 (Coleoptera: Tenebrionidae: Pedinini). Zootaxa 3399: 23–34

[B39] Kamiński MJ Raś M (2011) New status of the genus *Ectateus* Koch, 1956 with taxonomic notes on the *Ectateus* generic group (Coleoptera: Tenebrionidae: Platynotina). Annales Zoologici 61: 647–655. doi: 10.3161/000345411X622507

[B40] Kamiński MJ Raś M (2012) Catalogue of the melanocratoid Platynotina (Tenebrionidae: Pedinini). Annales Zoologici 62: 227–243. doi: 10.3161/000345412X652756

[B41] Kamiński MJ (2012) Taxonomy of the Afrotropical genera *Angolositus* Koch, 1955 and *Pseudoselinus* Iwan, 2002 with a key to species (Coleoptera: Tenebrionidae: Pedinini). Zootaxa 3500: 49–60 10.11646/zootaxa.3709.6.1 26240926

[B42] Kamiński MJ (2013a) Two new species of the Afrotropical genus *Quadrideres* Koch, 1956 (Coleoptera: Tenebrionidae: Pedinini), with notes on the *interioris* species-group. Annales Zoologici 63: 85–94. doi: 10.3161/000345413X666129

[B43] Kamiński MJ (2013b) Taxonomy and distribution of the Afrotropical genus *Anchophthalmops* Koch, 1956 with a key to species (Coleoptera: Tenebrionidae: Pedinini). Zootaxa 3709: 501–523. doi: 10.11646/zootaxa.3709.6.1 10.11646/zootaxa.3709.6.1 26240926

[B44] Kamiński MJ (2013c) A new genus and species of the Afrotropical Platynotina from Tanzania (Coleoptera: Tenebrionidae: Pedinini). Acta Entomologica Musei Nationalis Pragae 53: 703–714

[B45] Kamiński MJ Iwan D (2013) Taxonomy of the genus *Kochogaster* Kamiński et Raś, 2011 (Coleoptera: Tenebrionidae: Pedinini), with description of a second known species. Zootaxa 3669: 027–036 10.11646/zootaxa.3669.1.3 26312317

[B46] Kaszab Z (1951) Tenebrionides In: Monard A Résultats de la Mission zoologique suisse au Cameroun. Institut Français d’Afrique Noire-Centre du Cameroun, 1–3

[B47] Koch C (1956) Exploration du Parc National de l’Upemba. II. Tenebrionidae (Coleoptera, Polyphaga), Opatrinae, First part: Platynotini, Litoborini and Loensini. Bruxelles, 472 pp

[B48] Kulzer H (1963) Verzeichnis des Typenmaterials der Tenebrionidensammlung des Museums G. Frey. Entomologischen Arbeiten aus dem Museum G. Frey 14: 375–434

[B49] Lacordaire M (1859) Histoire naturelle des Insectes. Genera des Coléoptères ou exposé méthodique et critique de tous les genres proposés jusqu’ici dans cet ordre d’insectes. Tribu XXV. Pédinides. Paris, 5: 1–400 [226–243].

[B50] Maddison WP Maddison DR (2011) Mesquite: a modular system for evolutionary analysis. Version 2.75.

[B51] Matthews EG Lawrence JF Bouchard P Steiner WE Ślipiński SA (2010) 11.14 Tenebrionidae Latreille, 1802. In: Leschen RAB Beutel RG Lawrence JF (Eds) Handbook of Zoology. A Natural History of the Phyla of the Animal Kingdom. Volume IV – Arthropoda: Insecta. Part 38. Coleoptera, Beetles. Volume 2: Systematics (Part 2) Walter de Gruyter, Berlin, 574–659

[B52] Mulsant E Rey C (1853) Essai d’une division des derniers mélanosomes. Mémoires de la Société Agriculturale de Lyon (Ser. 2), 2: 226–329

[B53] Nixon KC (2002) WinClada, Version 1.00.08. http://www.cladistics.com

[B54] Olson DM Dinerstein E Wikramanayake ED Burgess ND Powell GVN Underwood EC D’Amico JA Itoua I Strand HW Morrison JC Loucks CJ Allnutt TF Ricketts TH Kura Y Lamoreux JF Wettengel WW Hedao P Kassem KR (2001) Terrestrial Ecoregions of the World: A new map of life on Earth (PDF, 1.1M) BioScience 51: 933–938. doi: 10.1641/0006-3568(2001)051[0933:TEOTWA]2.0.CO;2

[B55] Péringuey ML (1892) Third contribution to the South African coleopterous fauna. Transactions of the South African Philosophical Society 6: 1–94. doi: 10.1080/21560382.1889.9526255

[B56] Raś M Kamiński MJ (2013) X-ray microtomography as a tool for taxonomic investigations: prothoracic skeletal structure in some Tenebrionidae (Coleoptera). Annales Zoologici 63: 381–391. doi: 10.3161/000345413X669649

